# Laboratory validation and field usability assessment of a point-of-care test for serum bilirubin levels in neonates in a tropical setting

**DOI:** 10.12688/wellcomeopenres.14767.2

**Published:** 2018-11-23

**Authors:** Laurence Thielemans, Ahmar Hashmi, Dah Dah Priscilla, Moo Kho Paw, Tekel Pimolsorntong, Thatsanun Ngerseng, Bart Van Overmeire, Stephane Proux, François Nosten, Rose McGready, Verena I. Carrara, Germana Bancone

**Affiliations:** 1 Shoklo Malaria Research Unit, Mahidol-Oxford Tropical Medicine Research Unit, Faculty of Tropical Medicine, Mahidol University, Mae Sot, 63110, Thailand; 2 Neonatology, Cliniques Universitaires de Bruxelles - Hôpital Erasme, Université Libre de Bruxelles, Bruxelles, 1070, Belgium; 3 Mahidol-Oxford Tropical Medicine Research Unit (MORU), Faculty of Tropical Medicine, Mahidol University, Bangkok, 10400, Thailand; 4 Centre for Tropical Medicine and Global Health, Nuffield Department of Medicine, University of Oxford, Oxford, OX3 7BN, UK

**Keywords:** Neonatal hyperbilirubinemia, point-of-care, bilirubin, low resource setting

## Abstract

**Background:** Screening and monitoring serum bilirubin (SBR) in neonates is crucial to prevent neonatal hyperbilirubinemia (NH)-associated morbidity and mortality worldwide. A lack of resources is often a barrier for measuring SBR in developing countries. Reliable, cost-effective, easy to use point-of-care (POC) SBR tests are needed. This study aimed to evaluate the technical accuracy and usability of the Bilistick System (BS), a new bilirubin POC test, in a tropical setting.

**Methods:** This was a mixed-methods study, including laboratory validation of the BS, direct observation of technical procedures as performed by the midwives and midwives’ assessment of the device’s easiness of use through focus group discussions (FGD) and a self-administered questionnaire. The study was conducted in a field clinic of the Shoklo Malaria Research Unit along the Thailand-Myanmar border between January and December 2017.

**Results: **A total of 173 samples were tested at a median age of 4 days. BS generated an error message—providing no SBR readout—in 48.6% of the tests performed. For the tests that yielded a result, the correlation coefficient (95% CI) between BS and routine laboratory bilirubinometer SBR was 0.87 (0.77-0.93). The accuracy decreased with increasing haematocrit and at higher humidity (≥75%). Direct observation of the operators using the device and analysis of the focus group discussions and questionnaires indicated that the BS was considered easy to use and required limited training.

**Conclusions: **This evaluation showed that the BS, in its current formulation, does not provide reliable results for measuring SBR in a tropical, low-resource setting  but has acceptable usability features.

## Introduction

Neonatal hyperbilirubinemia (NH), a common disorder worldwide, has a benign course if promptly managed. However, in settings with limited access to diagnosis and care, there is a higher morbidity and mortality risk ^1^. Accurate testing for elevated serum bilirubin (SBR) in the first days of life, followed by appropriate treatment with phototherapy, is crucial to prevent brain damage in infants ^2, 3^. Serum bilirubin concentration is the gold standard reference for determining NH management ^2– 5^, and it is done by laboratory-based colorimetric assay, which requires large blood volumes and a fully-equipped laboratory—often beyond the reach of clinics in low-resource settings. While visual assessment by Kramer zone scores ^6^ poorly correlates with bilirubin concentration ^7– 9^, transcutaneous bilirubinometers (TcB) have proven to be an alternative to invasive blood sampling ^10– 12^. However, TcB may under- or overestimate SBR and, as they assess extravascular bilirubin, are unreliable if the neonate has received phototherapy treatment ^13– 17^. The need for postnatal age- and ethnicity-specific nomograms further complicate TcB use ^18– 20^.

Frontline health workers in low-resource settings need reliable and cost-effective point-of-care (POC) tests for early screening of neonates at risk, timely treatment or referral when phototherapy is not locally available and monitoring of neonates requiring treatment. A promising, low-cost device called BiliSpec has been field tested in Malawi, showing good agreement with the standard reference (Pearson’s correlation, r=0.97) and a mean difference of 5.1 µmol/l with 95% confidence interval between −29.1 and 37.6 µmol/l ^21^. An alternative POC device is the Bilistick (BS) system, which features a hand-held, battery-operated machine requiring a smaller blood volume and generating a faster result than the standard laboratory test. When compared with laboratory assays on duplicate plasma samples of 118 neonates in Italy and Egypt under ideal conditions, BS showed an acceptable mean underestimation of SBR levels of 10.3 µmol/l and a 95% confidence interval between −38.0 and 58.7 µmol/l ^22^. In 2017, in a tertiary care referral centre in Cairo, samples from neonates with SBR less than 340 µmol/l were used to investigate the accuracy of the BS on venous blood ^23^. The study concluded that BS had comparable accuracy to the TcB JM-103. A recent multi-centre study reported the BS to slightly underestimate SBR value with maximum limits of agreements of −128.3 to 102.6 µmol/l ^24^.

These findings suggest that new POC assays to diagnose NH have promise for use in low-resource settings, but there is insufficient evidence to make strong recommendations. The current study used a mixed-methods approach to evaluate two important features of the BS: (i) the technical accuracy of the BS in tropical field conditions, and (ii) usability of the device among locally trained staff in a low resource setting.

## Methods

### Outline and location

This prospective, mixed-methods study was conducted by the Shoklo Malaria Research Unit (SMRU) at the Wang Pha clinic, located along the Thailand-Myanmar border in the north-western province of Tak, Thailand. The study evaluated BS during two distinct periods: from the end of January until the end of April 2017, and from the beginning of June until the end of December 2017.

The field clinic and on-site laboratories have stable electricity, basic equipment and refrigerators. The staff consists of locally trained medics, midwives, nurses, health workers and laboratory technicians. Prior to starting the study, all midwives (n=13) and 3 laboratory technicians were asked and agreed to participate to the study. They were then trained on use of the BS system. The training module was created and conducted in English and in the local language (Sgaw Karen) by two authors (L.T. and D.D.P) based on protocols developed by the manufacturer; user instructions were simplified in a PowerPoint presentation for the local staff ( Supplementary File 1). The training consisted of two sessions: 1 hour of theory with a step-by-step description of the procedure supported by pictures and on-site demonstration, followed by 1 hour of practical, hands-on training.

The study included neonates at ≥35 weeks of gestational age in-born at SMRU clinics or out-born (mostly at home) who were brought to the clinic; in stable clinical condition, and requiring bilirubin measurement for either: (i) a grade 3 or higher jaundice observed visually as per the Kramer scale ^6, 8^, or (ii) a previous, borderline SBR measurement (i.e. ≤50 μmol/l below the treatment threshold of the British National Institute for Health and Care Excellence (NICE) guidelines ^3^). All parents of neonates who met the inclusion criteria were proposed to participate in the study. The study was explained in the preferred local language and the parents who agreed signed an informed consent form.

 Since the BS required a haematocrit (HCT) ≤65% to provide a reading, neonates with a prior HCT >65% or no prior HCT measured were not eligible. Each infant enrolled contributed to one measurement only.

Gestational age was defined by ultrasound at the first antenatal consultation ^25, 26^ or by Dubowitz gestational assessment at birth ^27, 28^. Neonatal age in hours and phototherapy treatment at time of SBR measurement were reported systematically.

### Technical validation

Simultaneously, two blood samples were collected by heel prick: one (50 μl) in a heparinized capillary tube for laboratory testing and one (25 μl) in a non-heparinized transfer pipette for BS.

The first 50-μl blood sample was used for the routine laboratory bilirubinometer SBR testing and transferred to the on-site clinic laboratory within 10 minutes of collection. The laboratory staff centrifuged the capillary tube (3 min at 10,000 rotations per minute) to separate red blood cells from plasma and estimate HCT using a Hawksley micro-haematocrit reader. To ensure quality, the accuracy of capillary HCT reading was assessed independently from this study. A total of 90 consecutive HCTs were independently read by two laboratory staff. The maximum observed difference was 11 HCT points; in 54% (48/89) of the samples, the two reading were the same, in 37% (33/89) the difference was 1 HCT point and in 9% (8/89) a difference of ≥2 HCT points was observed. There were no statistical differences between paired HCT (Wilcoxon matched pairs signed rank test, p=0.089).

In the study, after HCT reading, the same tube was used to measure SBR using the dual-wavelength BR-501 bilirubinometer (Apel Co., Ldt, Japan) according to manufacturer’s instructions. The BR-501 bilirubinometer has ± 5% accuracy within the measurement range (0–30 mg/dl), as reported by the manufacturer.

The second blood sample was used for the study device. The BS system (Bilimetrix srl, Italy) consists of a hand-held reflectance reader that uses test strips composed of a filter coupled with a nitrocellulose membrane ( Supplementary File 2 and http://www.bilimetrix.net/). The device is powered by rechargeable batteries. The BS system was ready to use after computerized installation and calibration (performed using a calibration set composed of 8 pre-calibrated strips provided by the manufacturer) according to the manufacturer’s instructions. The BS was set up by the manufacturer to show a weekly message advising calibration with a randomly selected strip from the calibration set. If the device reported an inadequate calibration on the single strip, then a complete calibration was done requiring the insertion of all 8 pre-calibrated strips twice. The calibration set had to be changed every 6 months and required computerized registration.

The BS reader was kept under ambient conditions for the entire duration of the study. The test strips were kept in their humidity-proof packaging and were exposed to ambient conditions for a few seconds prior to performing the test.

The test was performed according to the manufacturer’s instructions. Although the reader was designed for a single operator, the midwives decided to perform the test in pairs. One midwife inserted a test strip in the BS system as the second midwife collected the two blood samples via heel prick during the 38 seconds necessary for the device to determine the reflectance of the dry strips. The blood was loaded on the strip within 2 minutes of instrument calibration. Measurement was displayed within 100 seconds ( Supplementary File 2). If the blood was not loaded within 2 minutes of the strip calibration, the reader displayed a warning message, requiring the strip to be removed and 2 additional minutes for the reader to reset prior to accepting a new strip. The new strip required a new 38-second calibration as well and thus the increased time spent in calibration could potentially cause clotting of the blood in the non-heparinized pipette. After each test, a visual evaluation of the strip was performed by the operator to confirm saturation of the membrane. If the reader was unable to indicate an accurate result for the bilirubin level, it generated an error message displayed on the BS screen. As per approved protocol, neonates were not pricked a second time and the BS test was not repeated when clotting occurred or error outputs messages were displayed.

Temperature and room humidity were recorded with a temperature hygrometer at the time of the test. Humidity level was classified as either normal or high humidity (<75% or ≥75%, respectively).

### Usability assessment

The mixed-methods approach employed both exploratory and explanatory design and was used to evaluate the usability of the BS system. This included observation by laboratory technicians of technical procedures performed by the midwives and midwives’ assessment of the device’s ease of use through focus group discussions (FGD) and a self-administered questionnaire. The questionnaire and FGD took place after 6 months of using the BS. This mixed-methods approach also allowed for triangulation through independent qualitative analysis of FGD data by two investigators (L.T., A.H.), and mutual corroboration between observed technical errors and midwives’ evaluation of the device’s ease of use via questionnaire.

A total of three laboratory technicians evaluated each step of the process using an internal assessment checklist ( Supplementary File 3) and reported technical issues (e.g., blood clotting).

After providing written informed consent, midwives filled in a 5-point Likert scale, self-administered questionnaire that was developed based on the operating procedures of the user manual ( Supplementary File 4). The questions were translated into the local language (Sgaw Karen), back-translated, and finalized prior to conducting this component of the study. The 5-point Likert scale ranged from “Strongly disagree” (score 1) through “Neutral” (score 3) to “Strongly agree” (score 5).

After completing the questionnaire, midwives participated in FGD to explore their experiences working with the BS device ( Supplementary File 5). FGD guides and questions were developed based on investigator knowledge and information shared by the manufacturer. FGD guides were translated into Sgaw Karen, back-translated and finalized prior to conducting FGD. To promote an environment in which participants felt comfortable talking, participants were purposively selected to form groups similar in clinical experience. There were two focus groups conducted, one with six junior and senior midwives and one with seven assistant midwives. The FGD facilitator (M.K.P.) was fluent in Sgaw Karen, the preferred language of the midwives, and assisted by L.T. FGD were audio-recorded and transcribed directly to English by a translator unfamiliar and uninvolved in the study.

### Data analysis

Sample size calculations for technical validity of BS assumed a power of 80%, an effect size of 0.25 and α = 0.0125, yielding a sample size of at least 180 samples needed to detect a statistically significant difference in measurement compared to routine laboratory bilirubinometer via two-tailed paired t-test. Proportions were compared using chi-square test and Mann-Whitney U-test was used for comparison of medians. A Bland-Altman plot was used to graphically inspect differences between the two tests. NICE guidelines ^3^ were used for clinical interpretation of routine laboratory bilirubinometer SBR to diagnose NH and direct treatment as needed. Data were analysed using STATA/IC 14.0 (Stata Corp 2015, Version 14.1. College Station, Texas, StataCorp LP).

The results of FGD were analysed by two researchers (A.H., L.T.). L.T. reviewed the transcripts and clarified any outstanding questions with M.K.P. Initially, open coding was employed upon preliminary reading of the transcripts (L.T. and A.H.). Then, line-by-line inductive coding was performed by L.T. and A.H., independently. Mind maps were constructed to help reach consensus on codes and coding hierarchy. Each researcher independently performed thematic analysis based on initial coding to identify emergent themes. Discrepant coding between the two investigators was discussed until consensus reached and coding adjusted accordingly. Queries were then performed on the textual data to identify similarities and variations in the two FGD. Analysis was facilitated by NVivo for Mac v11.4.0 (QSR International, Melbourne, Australia).

In order to create an easy-to-use scale to analyse, two questions of the self-administered questionnaire (nr1 and nr9) had to be reversed. Scores were then described using median (IQR) and their distribution demonstrated by a histogram. Reliability of the questionnaire was assessed by the Cronbach alpha coefficient ^29, 30^.

### Ethical clearance

The study was approved by the Ethic Committees of the University of Oxford, UK (OXTREC 5115-16) and the Faculty of Tropical Medicine, Mahidol University, Thailand (TMEC16-073). The Tak Community Advisory Board, consisting of members of the local community, also revised and approved the study (TCAB-05/Rev/2016). All 13 midwives trained to perform the BS consented to the self-administered questionnaire and participate in FGD. Written informed consent was obtained from the parents or guardian of the neonates.

## Results

### Overview of validation data

**Figure 1.**
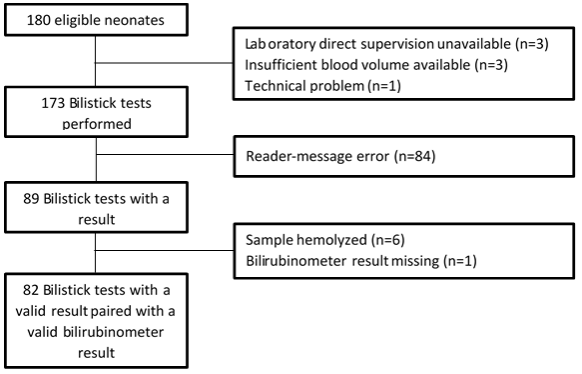
Study flowchart.

Consent was obtained from parents of 180 eligible neonates. In total, three tests were excluded from analysis because the laboratory technicians were unavailable to supervise the procedure; four additional tests were excluded from further evaluation: three had insufficient amounts of blood to complete the BS test, and the reader stopped functioning during one test, yielding an incomplete test result. Overall, BS was performed on 173 blood samples ( Figure 1).

Most neonates enrolled in the study were born at term (162/173, 93.6%); gestational age was estimated by ultrasound in 69.4% of the neonates (120/173). The majority were in-born at the SMRU clinic (160/173, 92.5%), the 13 out-born included 11 in a tertiary center, 1 at home and 1 in another clinic. The test was done at a median age of 4 days; the median (IQR) value of the last HCT prior to recruitment was 57 (54–61), ranging from 31% to 65% and the median (IQR) HCT value at recruitment was 55% (52–60). Eleven samples had a HCT value above 65%, the cut-off HCT to allow correct saturation of the BS strip membrane. Median room temperature at time of sampling was 28.9°C (range: 23–36°C) and humidity 67% (range: 39–89%). A total of eight neonates were under phototherapy treatment at the time of sampling ( Table 1).

**Table 1.** Baseline newborn and environment characteristics at time of bilirubin measurement.

Characteristics *	All (n=173)	BS result (n=89)	BS errors (n=84)	p-value
**Newborn**				
Gestational age, weeks	39 [38-40]	39 [38-40]	39 [38-40]	0.948
Age, hours	99 [63-161]	128 [70-170]	83 [56-146]	0.008
Haematocrit, %	55 [52-60]	53 [49-55]	59 [56-63]	<0.001
Under phototherapy, n (%)	8 (4.6)	3 (3.4)	5 (6.0)	0.429
**Environmental**				
Room temperature, °C	29 [28-30]	29 [28-30]	29 [56-63]	0.245
Room humidity, %	67 [58-77]	69 [58-78]	67 [57-76]	0.489

*Median [Interquartile range] unless otherwise stated.

### Technical support

Three different BS readers were received by SMRU and two readers were used over the course of this study. Reader #0 stopped functioning on January 6th 2017 prior to the start of the study and was replaced by reader #1 (25 January 2017). After 6 weeks (8 March 2016) reader #1 stopped functioning and was fixed via remote access by the Bilimetrix team in Italy. During the first two months of the study a high proportion of “reader generated error messages” was observed (29/51, 56.9%) which was attributed to the test strips nearing their expiry date. New strips were sent and used but by 30 April 2016, the proportion of “reader generated error messages” remained unchanged (22/39, 56.4%) which led the manufacturer to provide the study site with a new reader. The study resumed in June 2016 with reader #2, equipped with updated software. In August 2016, reader #2 stopped functioning and was fixed via remote access from Italy and performed normally until the end of the study (11 December 2016). Although significantly decreased with the latest device, the proportion of “reader generated error messages” remained high (33/83, 39.8%). Overall, both readers reported a total of 84 “reader generated error messages” (48.6% of all measurements).

### Technical validation

An error message was generated by the reader for the 11 samples above the HCT threshold of readability (>65%) and no SBR readout was available ( Table 2). Furthermore there was no SBR readout in 75% (54/72) of the samples with a HCT range of 56–65% and in 22% (19/87) of the samples with a HCT range of 41–55% ( Table 2).

**Table 2.** Proportion of error messages by neonatal haematocrit (HCT) categories.

HCT * range, %	All, n	Total errors, n (%)	reader #1 (n=90)	reader#2 (n=82)
31 ≤ 40	2	0 (0)	0/2 (0)	-
41 ≤ 55	87	19 (21.8)	9/42 (21.4)	10/45 (22.2)
56 ≤ 65	72	54 (75.0)	33/37 (89.2)	21/35 (60.0)
66 ≤ 75	11	11 (100.0)	9/9 (100.0)	2/2 (100.0)

*1 missing HCT. Proportion of error messages increased significantly with higher HCT (Chi square for trend p<0.001 overall, p<0.001 with reader #1 and p=0.005 with reader #2).

There were three types of reader-generated error messages encountered ( Supplementary File 6); EC:T06 (Uncomplete reading procedure within the established time), EC:B03 (Error identified during the initial phase of the bilirubin measurement) and EC:B04 (Error identified during the bilirubin measurement). The majority (64/84, 76.2%) were due to a failure of the reading procedure (described by the manufacturer as potentially linked to the HCT level). They were all found in equal proportion at different humidity levels (<75%, ≥75%, p=0.242), but the proportion of error “EC:B04” (i.e., insufficient test strip saturation) was significantly lower (p=0.004) with reader #2 ( Supplementary File 6). The proportion of reader-generated error messages did not differ significantly among different midwives (p=0.306) or over time (chi-square for trend per month, p=0.392) and was not associated with gestational age, room temperature or humidity ( Table 1). Of the 89 samples with an available SBR result, 7 were excluded from further analysis: the reader reported haemolysis for 6 tests and one had no laboratory bilirubin measured ( Figure 1). The remaining 82 BS measurements could be compared with routine laboratory bilirubinometer SBR results: 36 from reader #1 and 46 from reader #2. 

**Figure 2.**
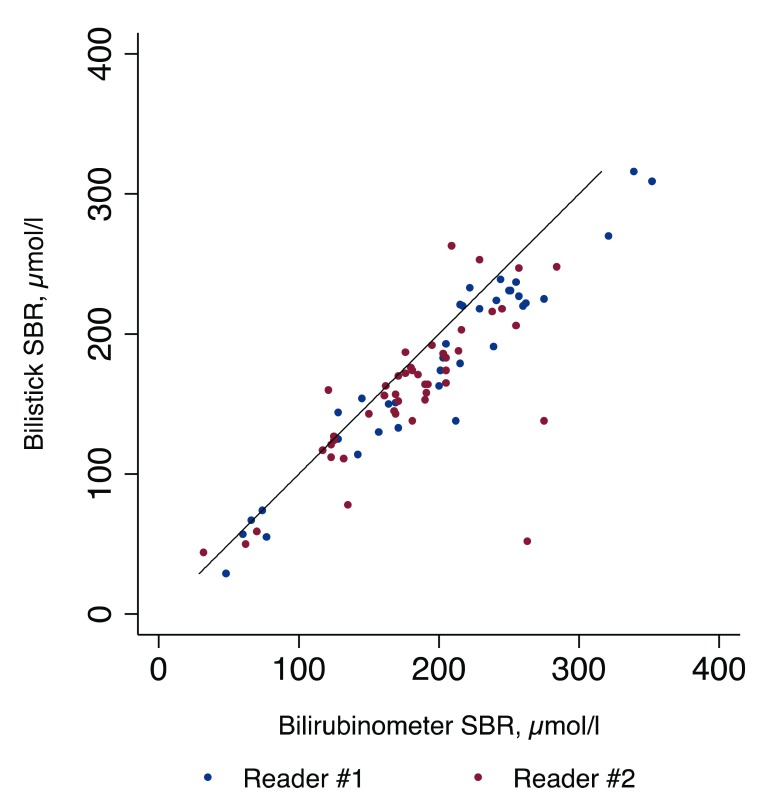
Correlation of bilirubin measurement between BS and routine laboratory bilirubinometer. Solid line is the y=x reference line. SBR, serum bilirubin. Blue dots are reader #1 and red dots are reader #2.

Reader #2 was mostly used during a period of high humidity (58.7% of the tests) and on samples with higher HCT ( Table 3). The correlation between BS and routine laboratory bilirubinometer SBR was higher for reader #1; the Pearson’s correlation coefficient (r) was 0.97 (95% CI: 0.93-0.98) compared to 0.71 (95% CI: 0.53-0.83) for reader #2 ( Figure 2). Taking the two readers together, this correlation varied depending on the ambient humidity; there was a high correlation at humidity levels <75% with a Pearson’s correlation coefficient r =0.96 (95% CI: 0.94 to 0.98), but it decreased to 0.62 (95% CI: 0.34 to 0.80) when measurements were done at high levels of humidity (≥75%). The HCT also influenced the BS measurement accuracy with a correlation coefficient of 0.95 (95% CI: 0.91 to 0.97) for the HCT range of 31-55% and 0.48 (95% CI: −0.04 to 0.80) for the HCT of 56−65%.

**Table 3.** Comparison of Bilistick System (BS) reader#1 and #2 from available results (n=82).

Variables *	All (n=82)	Reader #1 (n=36)	Reader #2 (n=46)	p-value
**Newborn characteristics**				
Gestational age, weeks	39 [38-40]	39 [38-40]	39 [38-40]	0.359
Age, hours	127 [70-169]	127 [82-221]	125 [68-168]	0.824
Haematocrit, %	53 [49-55]	50 [49-54]	54 [52-56]	0.046
**Environment characteristics**				
Room temperature, °C	29 [28-30]	28 [27-31]	29 [29-30]	0.782
Room humidity, %	69 [58-78]	58 [52-65]	76 [69-81]	<0.001
**Humidity level, n (%)**				<0.001
Normal	52 (63.4)	33 (91.7)	19 (41.3)	
High	30 (36.6)	3 (8.3)	27 (58.7)	
**Bilirubin measurement characteristics**				
BS bilirubin, µmol/l	168 [138-218]	187 [136-226]	164 [138-186]	0.266
Bilirubinometer bilirubin, µmol/l	192 [150-229]	214 [151-251]	181 [150-205]	0.045
PCC of bilirubin values between BS and routine laboratory bilirubinometer, r (95% CI)	0.87 (0.77-0.93)	0.97 (0.93 - 0.98)	0.71 (0.53 - 0.83)	

*Data presented as median [Interquartile range] unless otherwise stated. PCC, Perason’s correlation coefficient.

When the humidity and HCT conditions were combined, the highest correlation was found for the HCT range 30–56% at humidity levels of <75% (r=0.97 (95% CI: 0.88–1.00)) and the lowest correlation was at a HCT range of 56–65% at levels of high humidity (r=0.29 (95% CI: 0.03-0.65)) ( Table 4).

**Table 4.** Pearson correlation coefficient (r) under different humidity and haematocrit levels.

Haematocrit level	Humidity level, r (95%CI)			
	<75%		≥75%	
30-55%	n=45	0.97 [0.95-0.98]	n=22	0.86 [0.69-0.94]
56-65%	n=7	0.90 [0.48-0.99]	n=8	0.29 [0.03-0.65]

Under “ideal” conditions (humidity <75% and HCT between 30 and 55%) correlation coefficient was similar for both readers: Reader #1 (n=30, r=0.97 (95% CI: 0.93–0.98)) and reader #2 (n=15, r=0.98 (95% CI: 0.94–0.99)).

**Figure 3.**
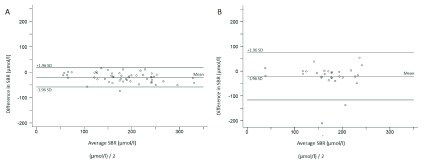
Bland-Altman plot for Bilistick System BS results as compared to routine laboratory bilirubinometer results. ( **A**) Humidity <75% (n=52); ( **B**) humidity ≥75% (n=30). Difference in SBR = (Bilistick SBR – Bilirubinometer SBR) (µmol/l), Average SBR = (Bilistick SBR + Bilirubinometer SBR) (µmol/l) / 2. SBR, serum bilirubin.

The Bland–Altman plot of the BS measurements (µmol/l) against routine laboratory bilirubinometer SBR performed at an ambient humidity of <75% (n=52) showed an acceptable mean difference of −20 µmol/l (limits of agreement −59 to 18). The maximum observed difference was −74 µmol/l ( Figure 3A). At ambient humidity levels ≥75% (n=30) there was a mean difference of −21 µmol/l with wide limit of agreement of −117 to 75 and a high maximum difference of −211 µmol/l ( Figure 3B).

### Usability assessment

***Direct observation of 173 sample measurements***. Midwives were consistently able to insert the strip into the reader, draw blood with the pipette, load blood onto the strips and read the results. The laboratory technicians reported a total of 9 major (5.2%) and 3 minor (1.7%) inaccuracies while observing midwives performing the procedure ( Supplementary File 3). The most frequent error was the formation of blood bubbles while loading the strip (n=9). All 12 observed errors happened within the first 3 months of the study (first month n=5, second month n=2, third month n=2); 10 of those errors led to a reader-generated error message ( Supplementary File 3), while the remaining two gave valid results (no haemolysis was reported by the reader).

Blood clotting was reported in two instances when the blood was not loaded within 2 minutes of the strip calibration, leading to the need of performing a new calibration and thus a delay in reading. Both tests led to a reading error message ( Supplementary File 6).

***Focus group discussion***. Emergent themes included: knowledge acquisition, midwife experience around training and use of BS; the clinical potential and considerations in implementing the BS at a larger scale; and midwife report of parents’ experiences.

Midwives knew the importance of screening for and diagnosing NH. The overall impression of midwives was that the training was simple, easy to understand, and greatly enhanced by the use of pictures. In fact, midwives thought that easy mastery of the instrument indicated that BS was appropriate to use in more rural and remote settings provided health workers received appropriate training.

*‘If they [village health workers] are familiar and can use it well, they can use it. You can also train the health worker to use the device because it is easy to use.’*—Assistant midwife

However, the midwives expressed confusion by the way results were displayed (in two units: µmol/l and mg/dl) as they were only familiar with the µmol/l results.

*‘If the result comes up, it is not the same as the lab result…. We know how to look at the lab result, but with the BS, how can we look at? … How can we look for the result in using BS because there are many numbers?’* —Senior midwife

Furthermore, midwives frequently commented on the device’s easiness of use. Another positive finding was the immediacy with which results could be obtained, often cited as a benefit compared to long wait times associated with the routine laboratory bilirubinometer testing.

*‘In my point of view, the BS is working faster than the machine used in the lab. When we send to the lab, we have to wait a little bit. And if there are a lot of patients and we are busy, we have to wait even longer*.’—Junior midwife

However, the bulk of the discussion around general use of the device centred on the frequency of errors. Indeed, this reflected a widely-held frustration of midwives using this device.

*‘We do as she [L.T.] teaches us but the result doesn’t appear on the device. Sometimes the result appears on the device. So I have no idea with this—is it because of what we did or because of the device? I don’t understand.’*—Assistant midwife

In the context of result reliability, settings appropriate for BS use were frequently discussed and often reflected training considerations. Midwives asserted that frequent errors limited the use of the device to facilities with adequate laboratory support. However, if results could be ensured, the speed with which the BS device could produce an accurate SBR measurement made the device favourable for use in low resource settings.

*‘We can use it without needing a lab if error does not appear. We just worry: if an error appears, we’ll have to start [the procedure] again. Without a lab, how do we do? So we need a lab.’*—Assistant midwife

*‘If we use the lab, we have to look for the result. But the device has automatic answers appearing in numbers so that we don’t need to look for the result.’* —Senior midwife

Finally, midwives asserted that the speed of diagnosis translated into prompt treatment of NH, with clear benefit for patients in low resource settings.

*‘[The device is]* g *ood for the children. If the children need phototherapy, we can give them in time before it gets worse. We can prevent severity.*’—Senior midwife

Parental concern arose as an important theme in discussions with midwives. Midwives reported that parents expressed concern and fear of heel pricks that may cause the infant pain. Thus, midwives would worry about having to repeat the tests due to BS error and often had to spend time counselling parents on the importance of heel pricks or repeat heel pricks for accurate diagnosis of NH. One assistant midwife mentioned that the parents of a baby, having witnessed multiple heel pricks, became increasingly wary of causing the infant undue pain leading them to avoid even routine immunizations.

‘[ *The parents] have compassion on the children when they see that the prick hurt their children. But parents don’t accept if [midwives] repeat the prick too many times. There’s a time that they tell us...It happens that they [parents] rejected vaccination because their children had been pricked so many times’—Assistant midwife*

***Usability questionnaire***. All thirteen midwives completed the questionnaire. Supplementary File 7 shows the distribution of participant’s answers on a 5-point Likert scale. The midwives agreed on the ease of use, with a median (IQR) score of 4 (4-4). The two questions that rated low were the only two negative statements; one about the training, *“I did not need to learn a lot of things before I could get going with the Bilistick system*” and one on the ability of the midwife to perform the test independently, *“I don’t need the support of a technical person (lab technician) to be able to use properly the Bilistick system”*. The results on these two statements were not in line with the midwives’ experiences assessed by FGD and it was suspected that the construction of the question as a negative was misunderstood. Cronbach alpha coefficient for Likert scale questions inter-reliability was low (0.5) with all statements included, but increased to acceptable internal reliability levels of 0.8 after removing the two negative statements. The median score remained the same after the two negative statements were removed. 

## Discussion

This study evaluated the hand-held bilirubin reader BS system developed to measure and display SBR concentration, for technical accuracy and usability in a low-resource setting ^23^.

The studied population was healthy near-term and term neonates in their first days of life (median age 4 days) with a high median capillary HCT of 55%. The young postnatal age and the use of capillary blood in the current study may explain the high HCT levels observed ^31, 32^. Moreover, delayed cord clamping is regularly practiced at the SMRU clinic according to WHO recommendations ^33^ resulting in newborns having a median HCT of 59% (IQR, 54-64) at 24 hours of life ^34^, a value similar to other settings worldwide ^31, 32, 35^.

This population contrasts with that of the three previous studies that tested the BS under ideal conditions: in the first study, neonates had a lower mean HCT value of 41.5% ^22^, those enrolled in the second study were of an older median age of 6 days with venous blood ^23^ and in the most recent multi-centre study, the median HCT was 42.7% IQR (38.0-48.0) at a median age of 4 days ^24^.

The current study detected a clear limitation of the readers in analysing SBR levels starting from HCT range 41–55% and major limitation in the HCT range 55–65%. Greco *et al*. documented a proportion of 6.8% (11/161) of technical problems using venous blood in older neonates ^23^, while the current study experienced 48.6% of “reader-generated error messages”. Overall, the device in its current formulation would not be suitable for screening in the early hours of life when mean HCT often ranges from 55 to 60% ^31, 32, 35^ and in settings where capillary sampling is used. The limitation of high HCT might have been underestimated from this study as neonates with previous HCT >65% were ineligible. Birth cohort ^34^ data from the same setting suggests the device would not be suitable for up to 1 in 4 neonates with HCT >65% between 2h to 30h of life: capillary Hct was >65% in 17.4% (193/1111) and Hct was ≥70% in 8.4% (93/1111) of the newborns (unpublished data, L. Thielemans).

For the tests that yielded a result, the two readers used for this study had performed similarly under ideal condition (humidity <75% and HCT between 30 and 55%) and the accuracy of both BS readers with humidity <75% were similar to those already published, showing comparable measures of the BS system by non-invasive rapid transcutaneous bilirubinometers ^23^. However, the BS accuracy was reduced with increasing HCT value (>55%) and higher humidity (≥75%). Had the BS test been used for NH diagnosis and management, only one neonate would have met the criteria for phototherapy according to NICE guidelines, compared to the five neonates diagnosed by routine laboratory bilirubinometer SBR. Given its lack of utility in younger neonates and its inaccuracy at high humidity and HCT levels, this study concludes that BS in its current configuration is not reliable for early screening of NH in this tropical setting.

This technical evaluation had some limitations: the BS was not compared to the gold standard measurement of serum bilirubin by HPLC ^36^ or with the Cobas c111 machine which would have required a larger volume of blood with venous sampling and analysis at the central laboratory in Mae Sot (30 km away). Moreover, due to the limited number of strips available, the high rates of errors, and the use of two different BS readers, the number of samples analysed in similar environmental conditions was small. At the time of manuscript submission, the BS manufacturer has already changed some features of the test strips and reports proper performances with blood sample with HTC up to 70%; further investigation will be required to assess the performances of the new device.

Assessing usability through both exploratory (observation) and explanatory (FGD, questionnaire) methods proved a strong approach. It confirmed that not only was the absence of SBR readout frequent, but that it gave the user considerable anxiety. Importantly, direct observation by laboratory technicians highlighted the small number of errors performed by the midwives when performing the BS test. Therefore, the assertion by the midwives that the device was easy to use, required limited training, and could be mastered in more remote settings is valid. However, the data from the usability assessment support the technical validation: the system proved to be poorly suited for low resource settings ^37^. More specific caveats in the use of this system require particular attention. The device required a level of technological capacity often beyond the means of low-resource settings ^37^. For example, the device requires computerized installation, registration prior to use, and—if technical assistance is required—remote internet access with compatible software. In addition to these technological constraints, one must consider the local capacity in information technology support to troubleshoot as problems arise. Secondly, the test itself required metered blood collection with a specific pipette, prompt loading of the strip as the pipettes are non-heparinized, and recalibration between each sample. Very strict time constraints are impractical in settings where the operator needs to sample blood from a newborn in a busy clinic. Although midwives reported the test to be easy to use, they spontaneously started performing the test in pairs in spite of its single-operator design. In addition to these clinical concerns, perceived infant discomfort of heel pricks led some parents to be sceptical of SBR testing; repeated sampling in a short time, because of close monitoring for borderline SBR values, in apparently healthy babies can be perceived as very distressing to the parents. This indicates the need to find a less invasive bilirubin screening tool for this setting.

Improving the performance under high humidity and with higher HCT levels, possibly with an improved strip matrix, could potentially make the BS system suitable for tropical settings. Heparinized pipettes, longer times for loading the sample on the strip or alternatively, collecting blood from a heel prick directly onto the strip, may improve the usability of the system. Minor changes such as results being displayed for a longer period of time and options to select preferred measurement units would benefit the system.

In conclusion, despite its innovative approach and easiness to use, the BS tested at SMRU is not suitable for SBR measurement in tropical, low-resource settings.

## Data availability

Due to ethical and security considerations, the data that supports the findings in this study can be accessed only through the Data Access Committee at Mahidol Oxford Tropical Medicine Research Unit (MORU). The data sharing policy can be found here: http://www.tropmedres.ac/data-sharing. The application form for datasets under the custodianship of MORU Tropical Network can be found in Supplementary File 8.

## Abbreviations

BS: Bilistick system

CI: Confidence interval

FGD: Focus group discussion

HCT: Haematocrit

IQR: Interquartile Range

NH: Neonatal hyperbilirubinemia

NICE: National Institute for Health and Care Excellence

POC: Point of care

SBR: Serum bilirubin

TcB: Transcutaneous bilirubinometers

SMRU: Shoklo Malaria Research Unit

WHO: World Health Organization

## Supplementary material

Supplementary File 1. Training material.

Supplementary File 2. Schematic representation of the (A1) Bilistick System and (A2) Bilistick display. *(Figures have been reproduced with permission from the Bilistick user manual)*.

Supplementary File 3. Procedural errors made by midwives under observation by the supervising laboratory technician. An internal Bilistick assessment checklist was used.

Supplementary File 4. The 5-point Likert scale self-administered questionnaire for midwives to assess the easiness of use of the Bilistick System.

Supplementary File 5. Focus group guide used with midwives to assess the usability of the Bilistick.

Supplementary File 6. Numbers, types and descriptions of BS generated errors accrued.

Supplementary File 7. Distribution of 13 participants’ answers on the easiness of use of the Bilistick System.

Supplementary File 8. Application form required to access raw data from this study.
