# Supplementary material for: Laboratory validation and field usability assessment of a point-of-care test for serum bilirubin levels in neonates in a tropical setting
Source: Wellcome Open Res. 2018 Nov 23;3:110. Originally published 2018 Sep 4. [Version 2] doi: 10.12688/wellcomeopenres.14767.2 (PMC6137410; doi:10.12688/wellcomeopenres.14767.2)

Bilistick training

Shoklo Malaria Research Unit

Wang Pha Clinic

Dah Dah Priscillia

Laurence Thielemans

Before to start

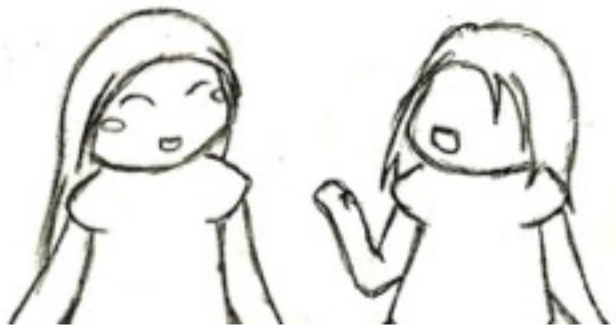

Drawing downloaded from http://konona-network.id.st

- Explain the procedure to the parents, answer questions
- Keep the neonate warm and quiet

How? swaddling the neonate in a cloth or blanket

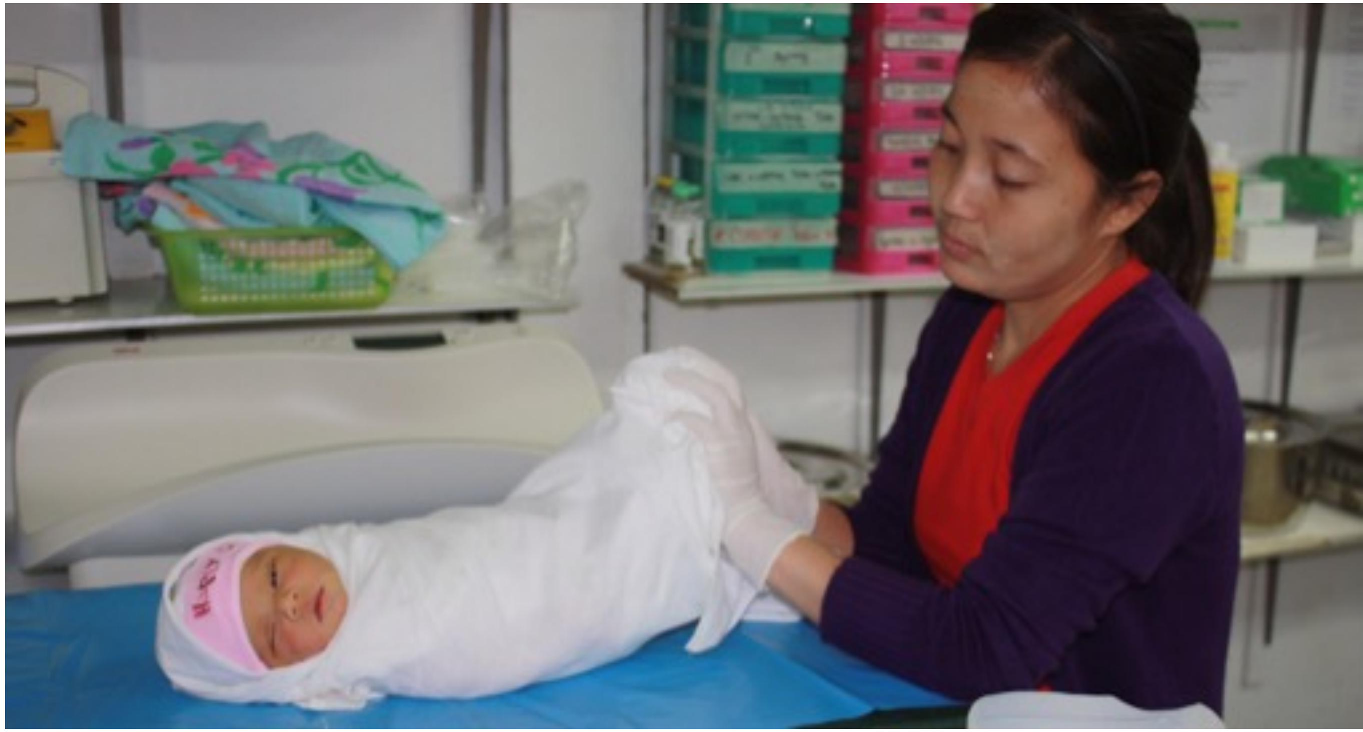

Credit Laurence Thielemans

Before to prick the baby

1. Put the **reader** on the table

2. Turn on the **reader** and wait → screen has to read : “***Ready to Insert strip***”

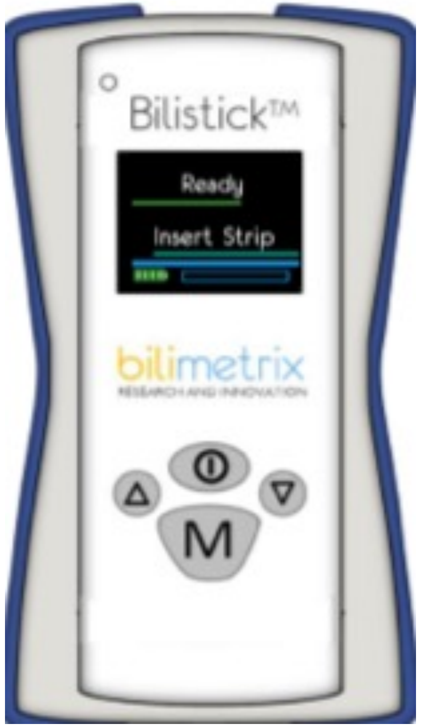

3. Prepare the **strip**:

   Open the package

   Do not touch the white parts.

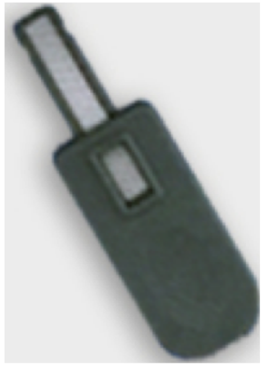

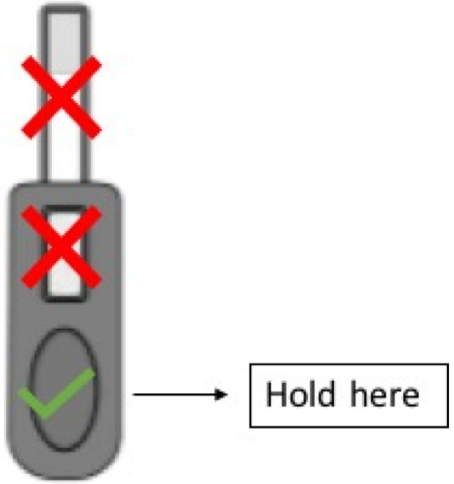

4. Get one **pipette** ready

Pictures from the Bilistick user manual edited

5. Wash hands and put on gloves

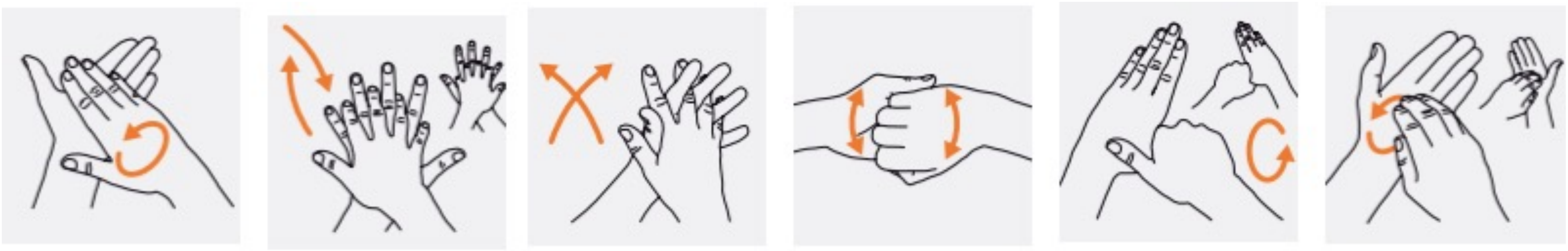

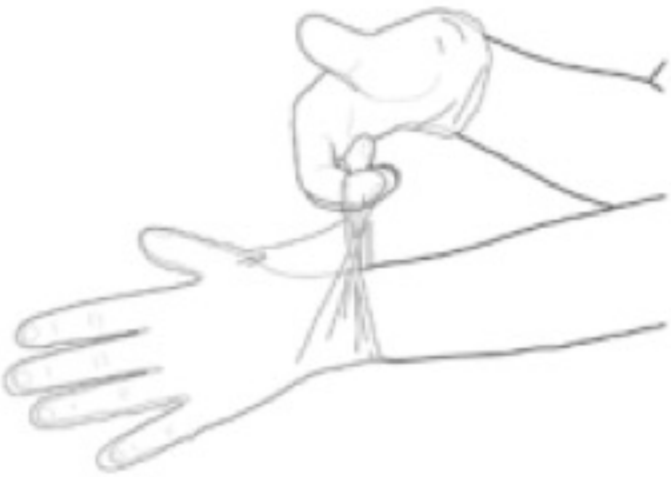

Pictures from WHO, Hand Hygiene: Why, How & When? And Glove Use Information Leaflet http://www.who.int/gpsc/5may/resources/posters/en/

Are you ready? → Prick the baby

Yes

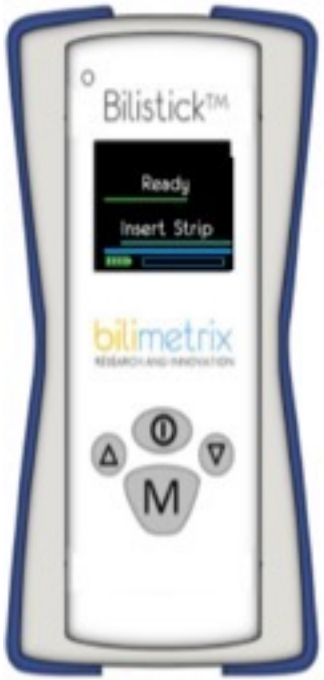

Bilistick Reader

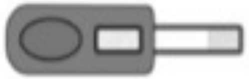

Bilistick Test Strip

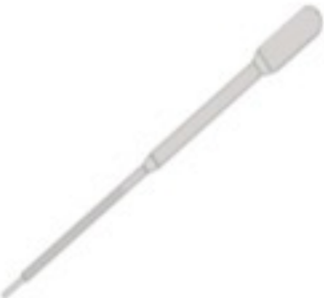

Bilistick Sample Transfer Pipette

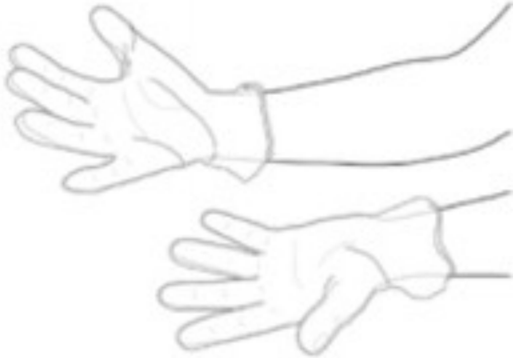

Remember: heel prick

How to hold the foot ?

Grasp foot exposing heel between thumb and index finger

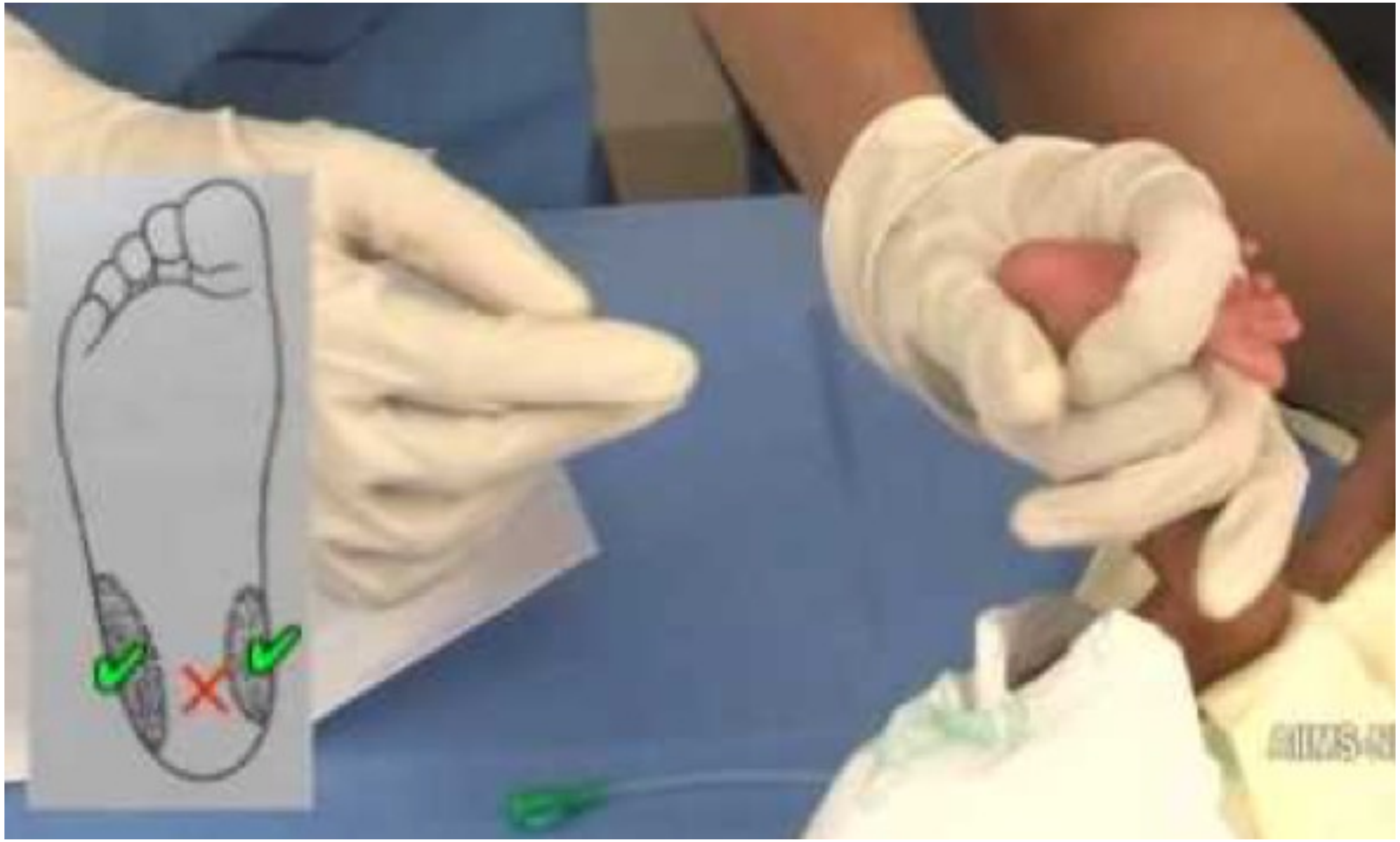

Picture exfrom the video clip of WHO Collaborating Center for Training and Research in Newborn Care AIIMS, New Delhi: heel prick for glucose estimation 2013

Heel Prick procedure

1. Disinfect and and let air dry

2. Puncture

3. Allow heel to recover for a few seconds

4. Wipe first drop of blood

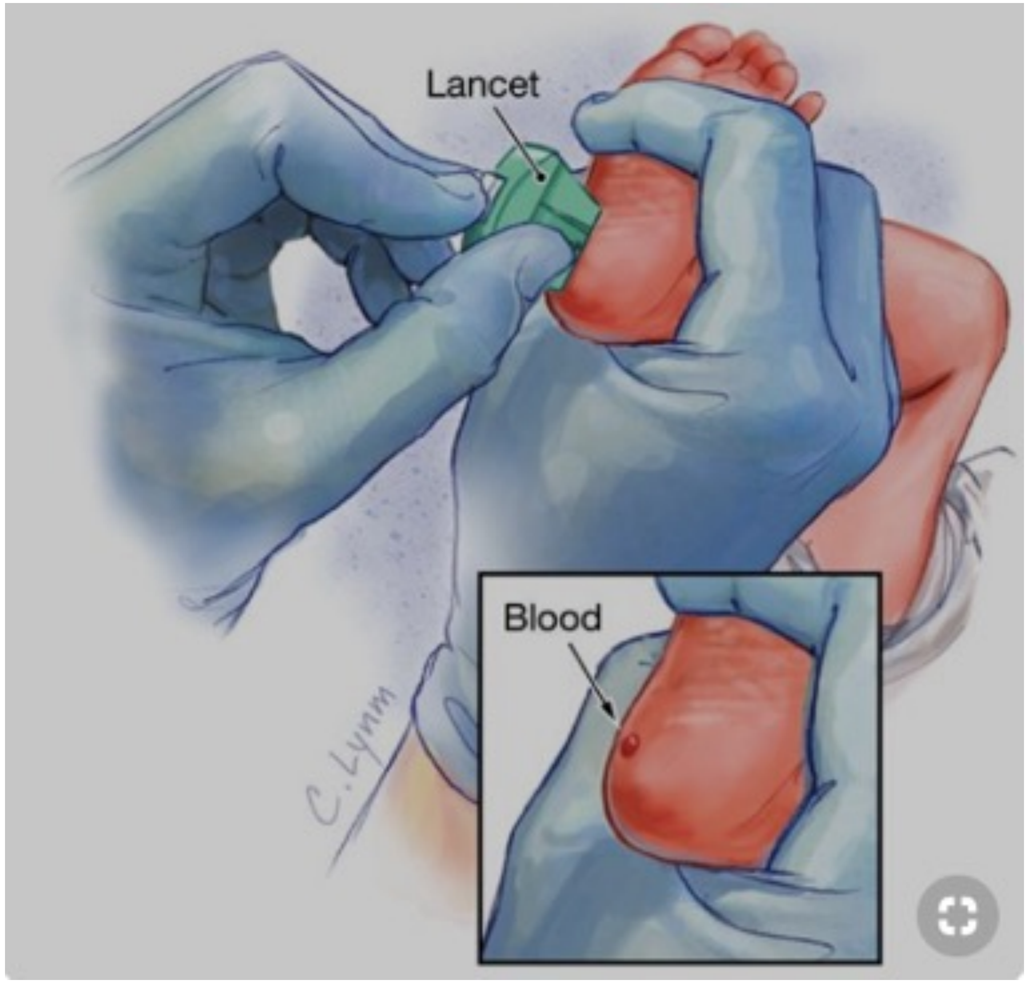

Picture from the JAMA network

Are you ready? → Prick the baby

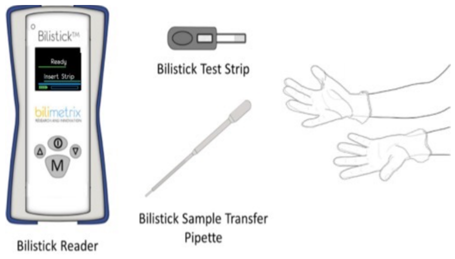

Good blood flow

Insert the strip

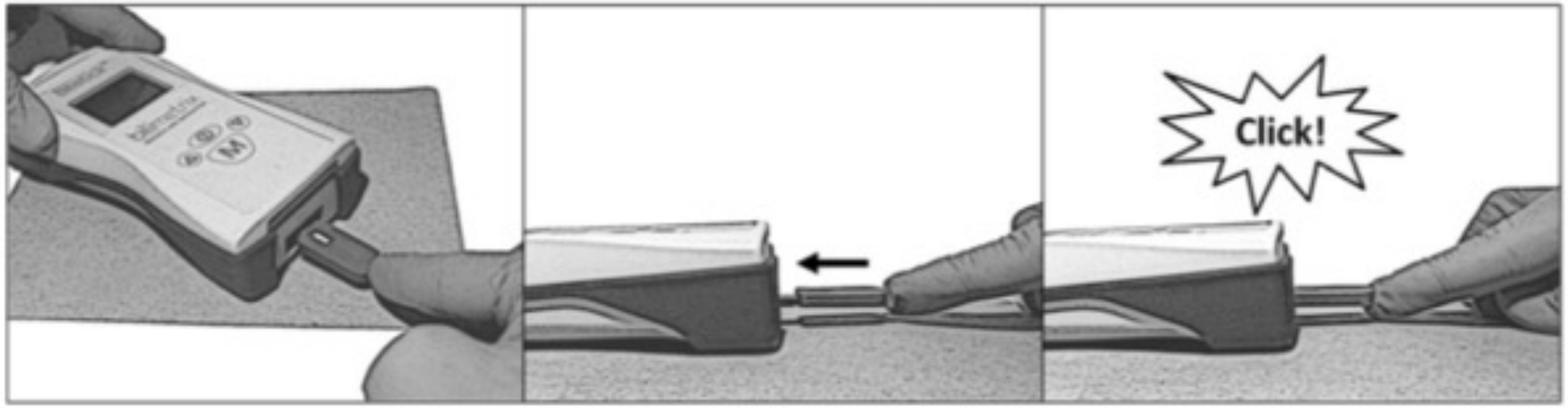

And wait

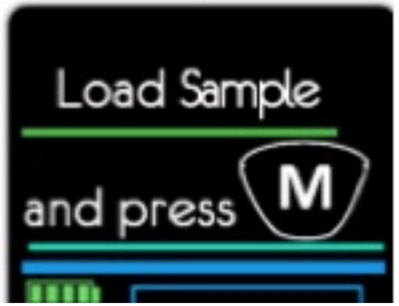

Ok to load the blood on the strip

Picture from the Bilistick user manual and instruction poster

How to hold the pipette?

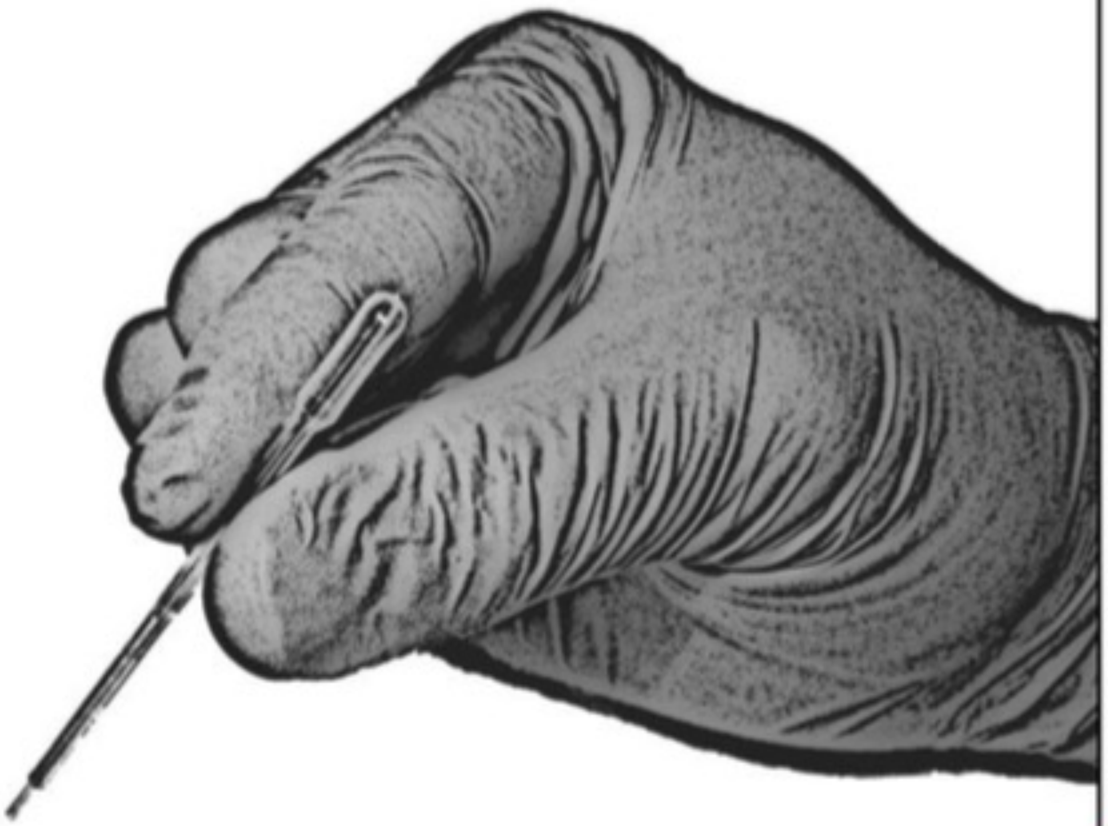

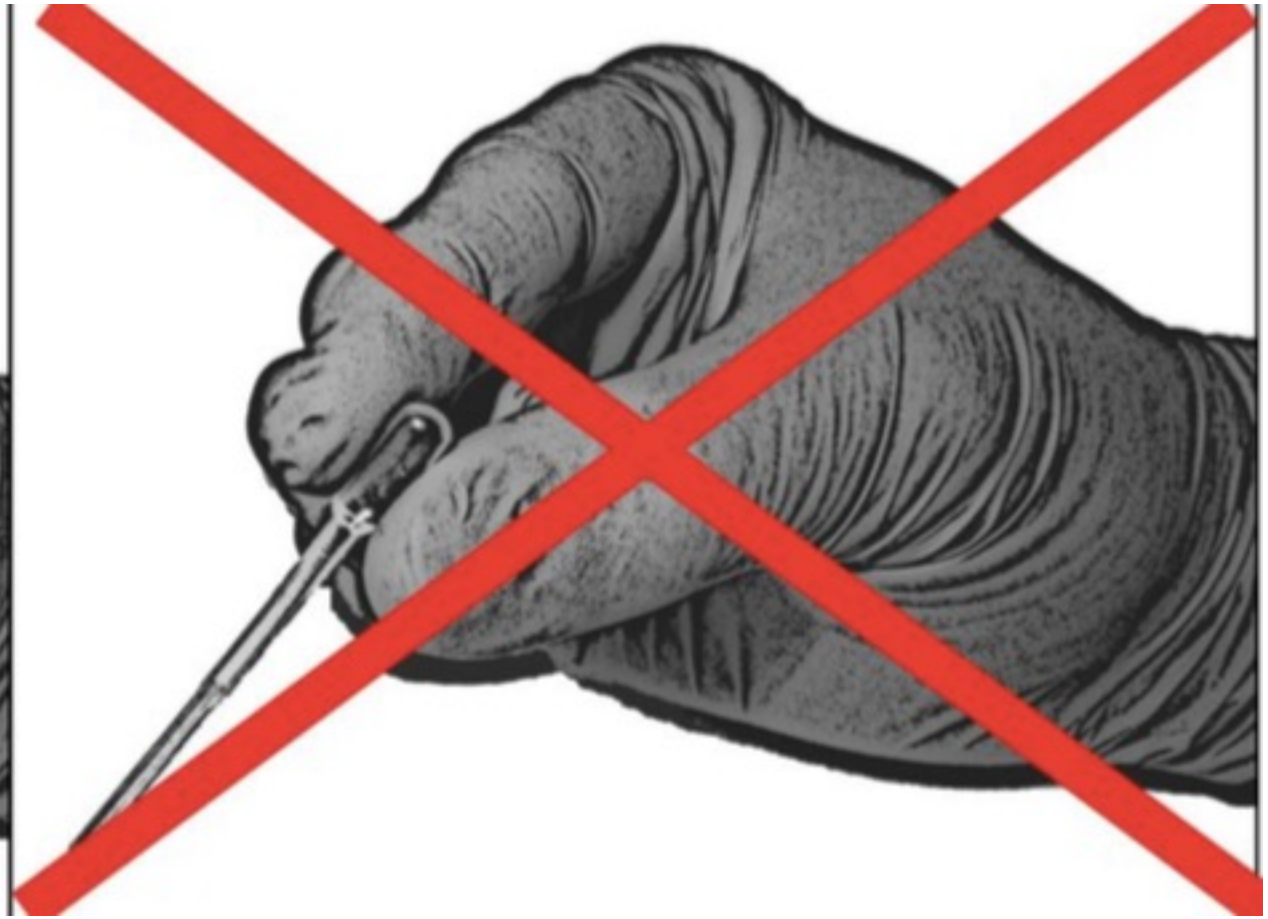

Do not hold the pipette by the bulb.

Picture from the Bilistick instruction poster

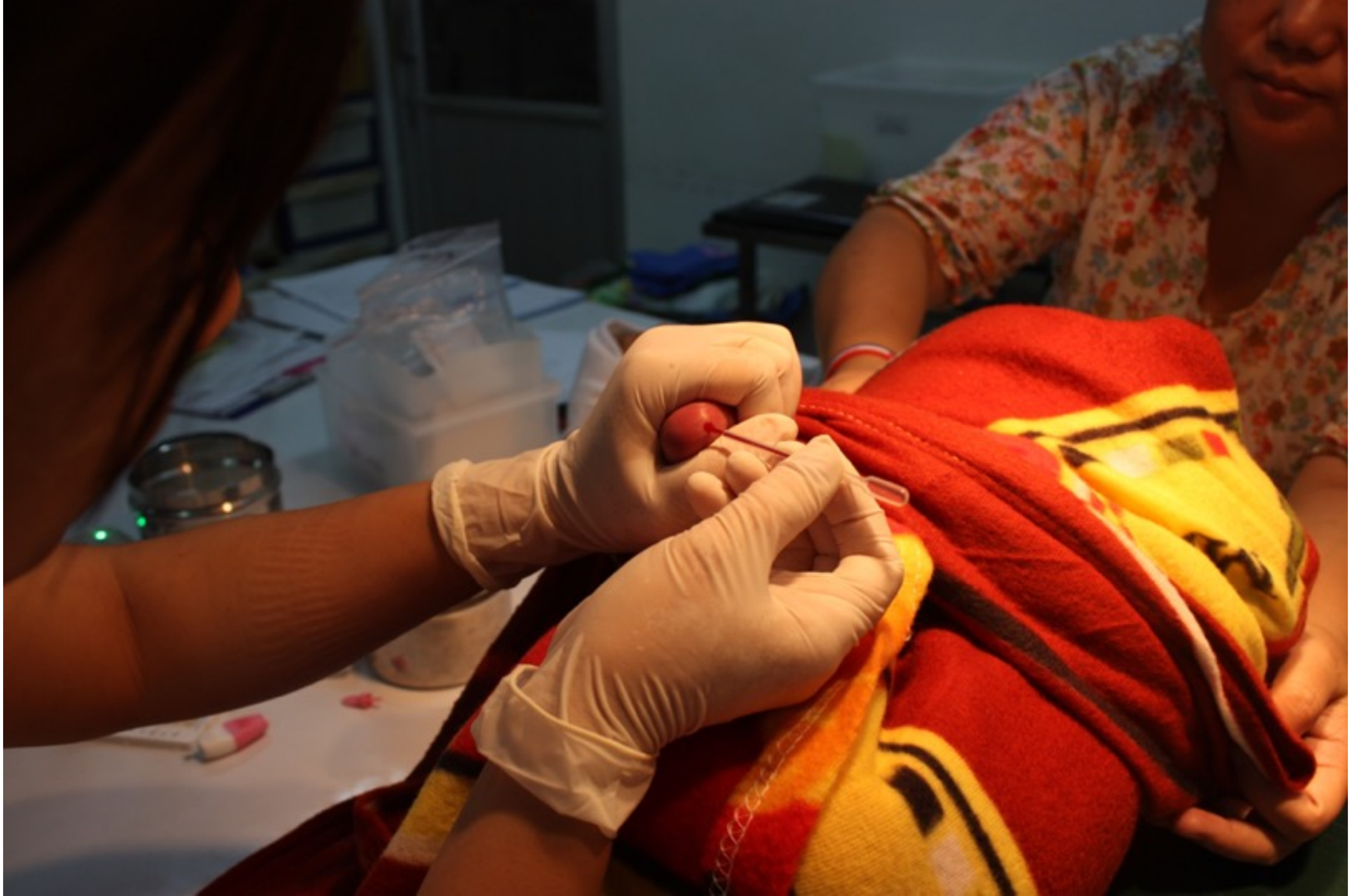

Credit picture: Laurence Thielemans

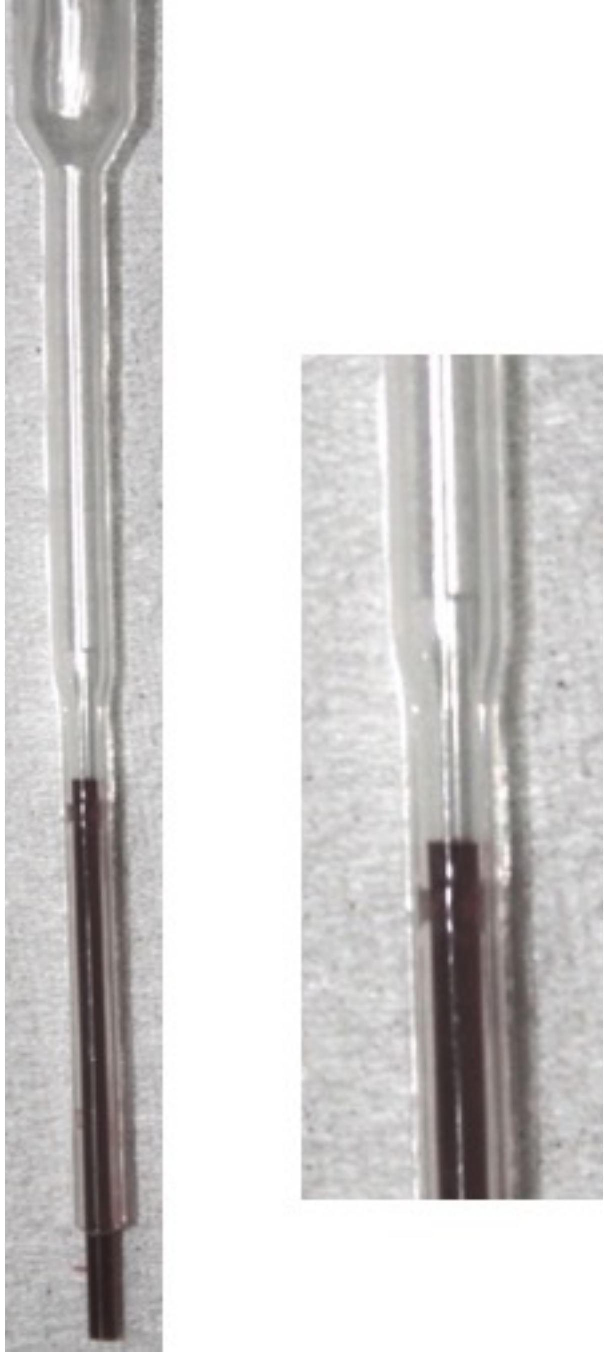

80% loaded

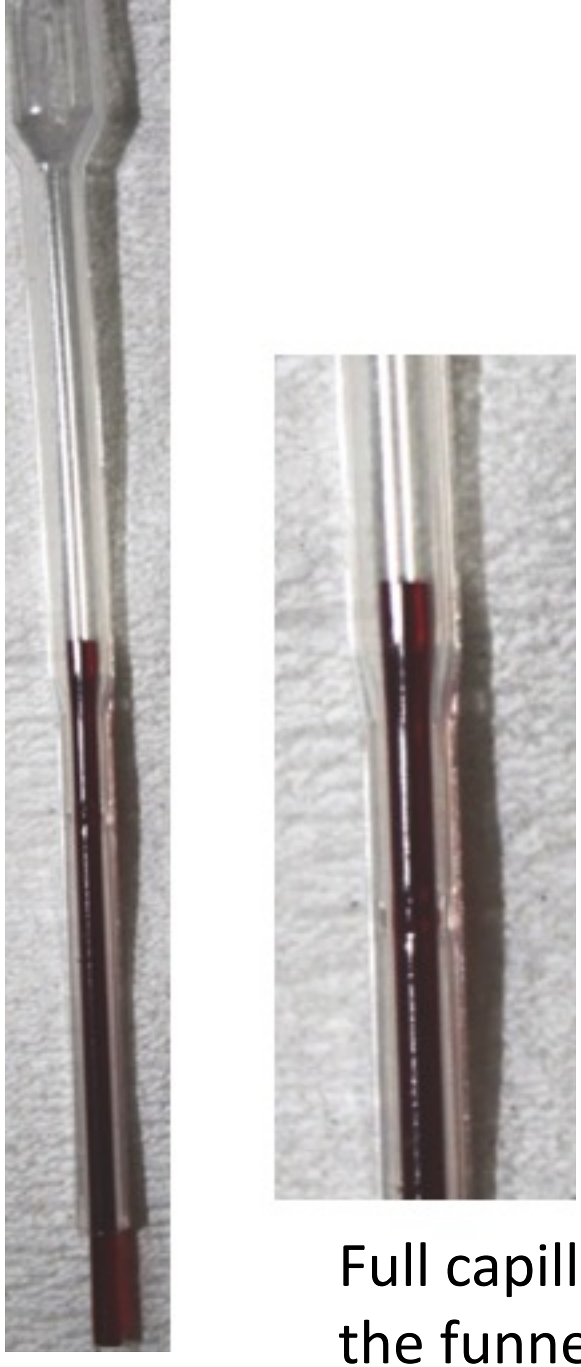

100% loaded

Full capillary tube: go above the funneling of the pipette

Credit Laurence Thielemans

How to load the blood on the strip?

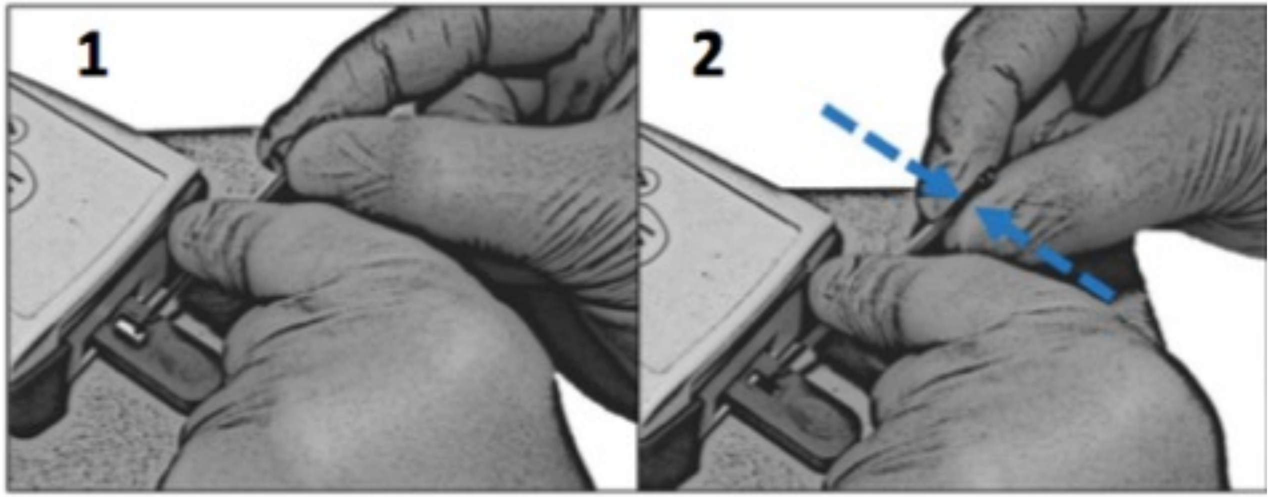

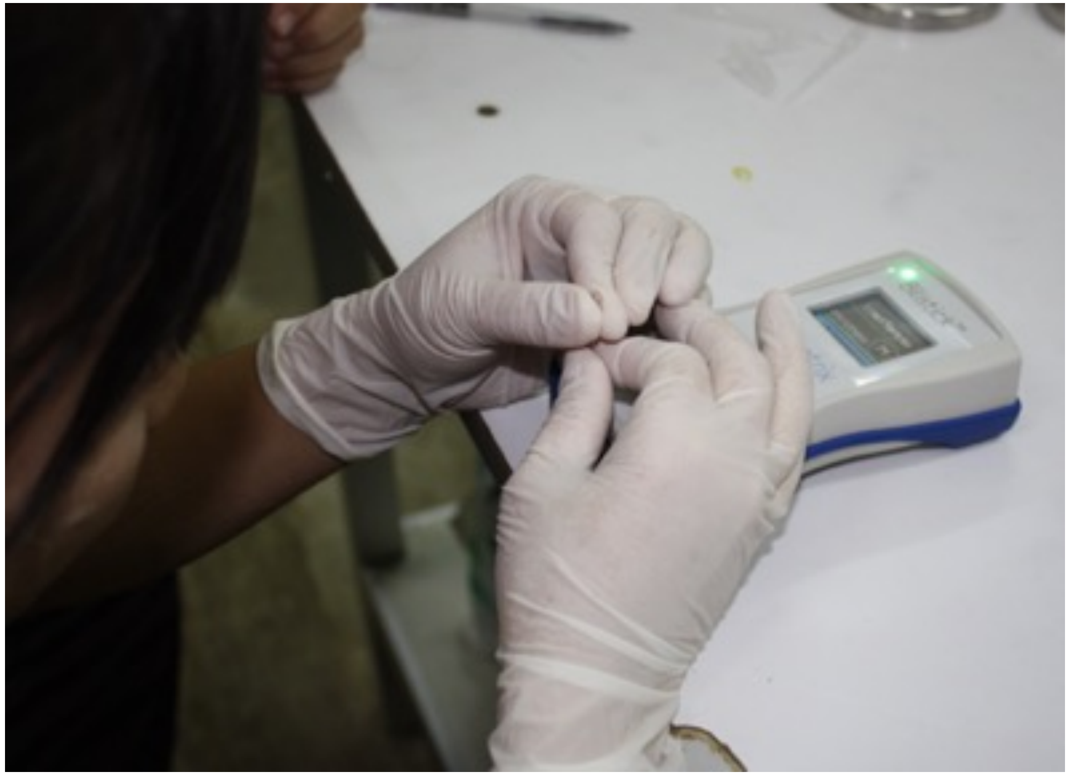

Credit : Laurence Thielemans

Slowly

! No bubbles!

Pictures from the Bilistick instruction poster

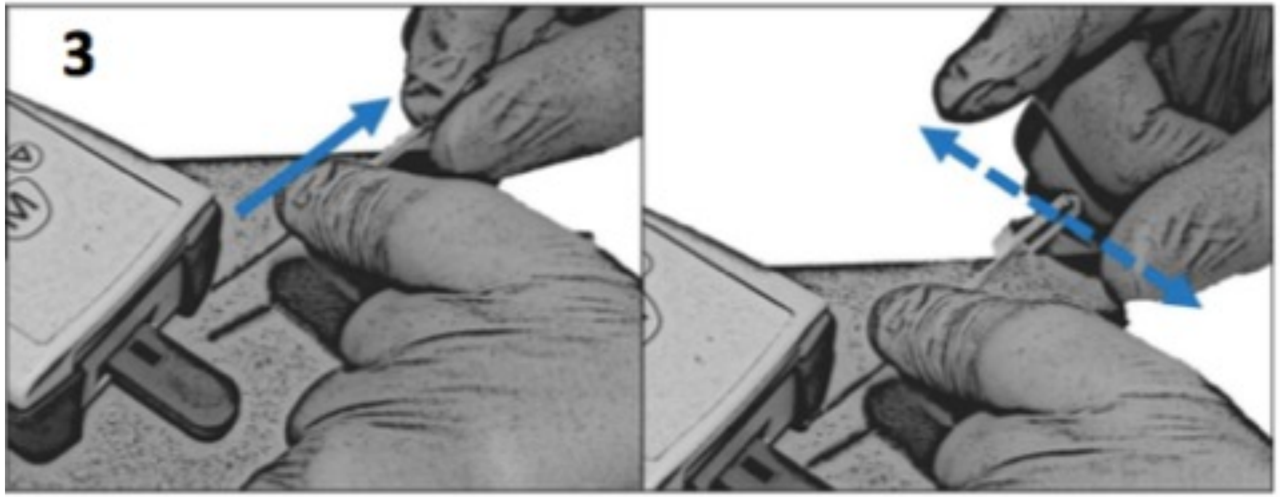

Press M

wait for the result

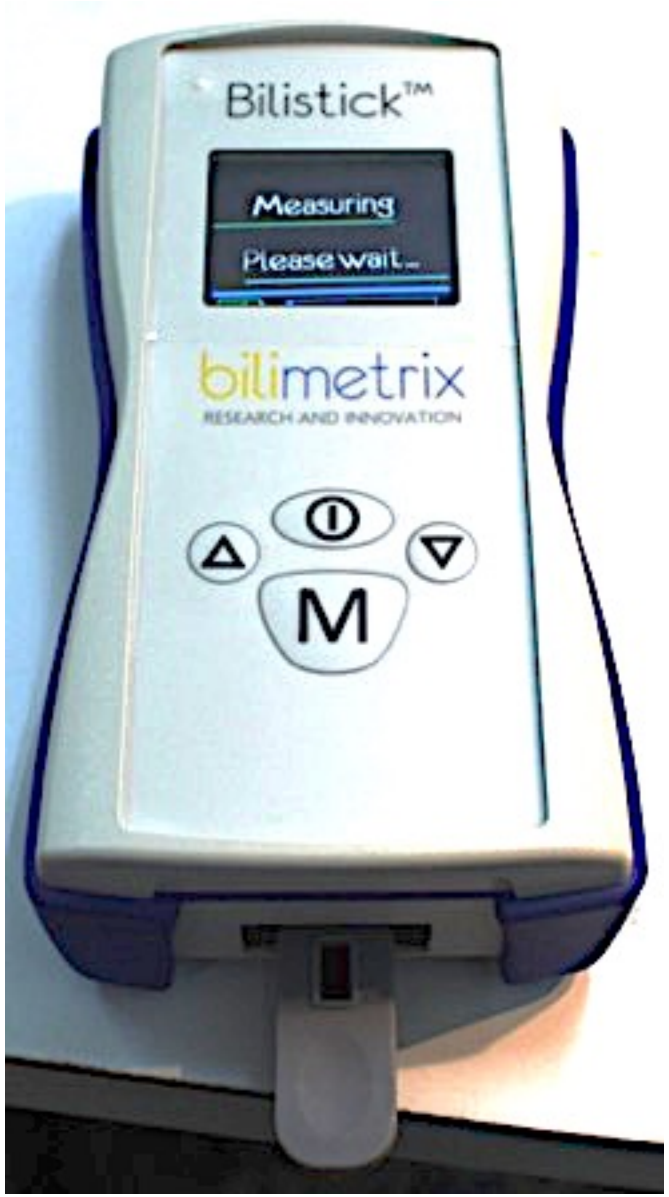

Credit Laurence Thielemans

Picture from the Bilistick instruction poster and edited

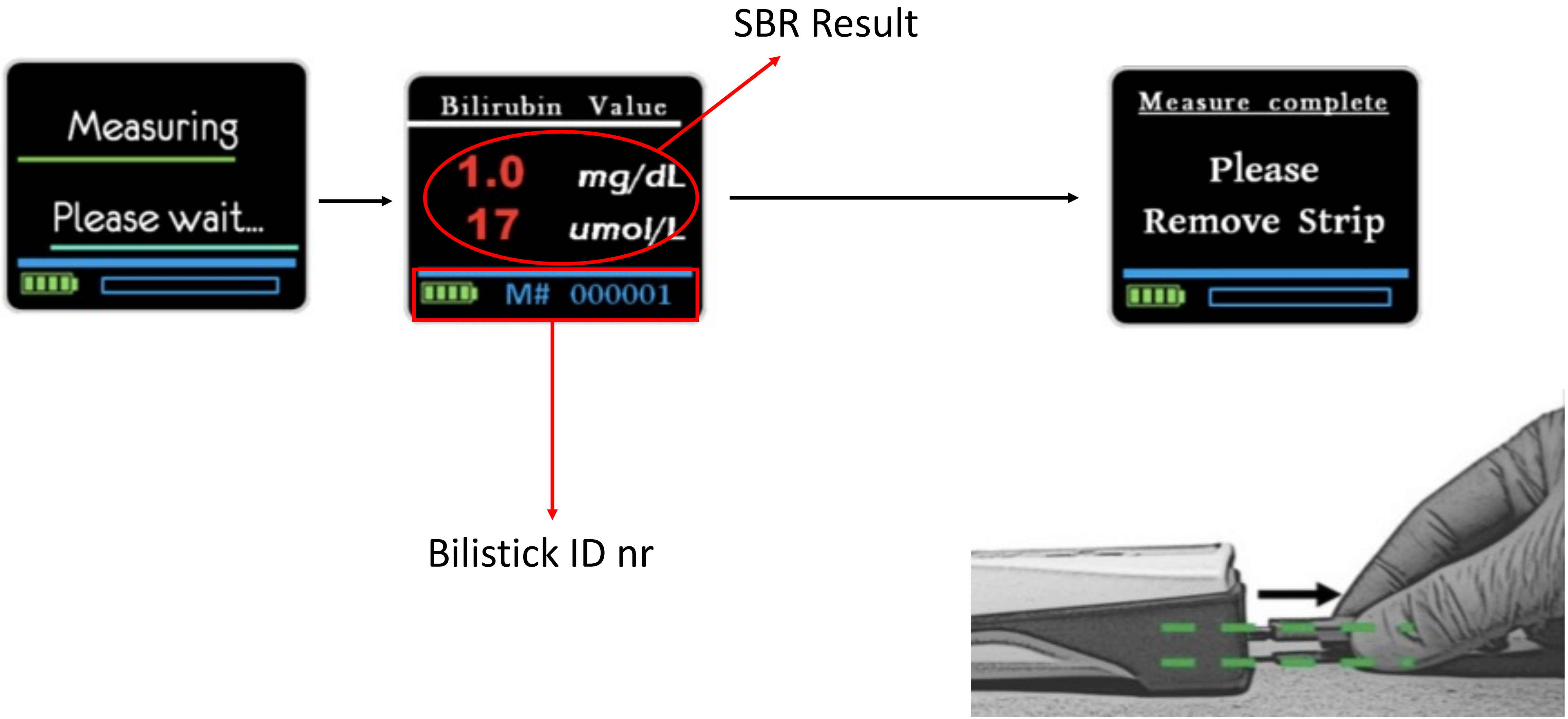

Picture from the Bilistick instruction poster and edited

Are you ready? → Prick the baby

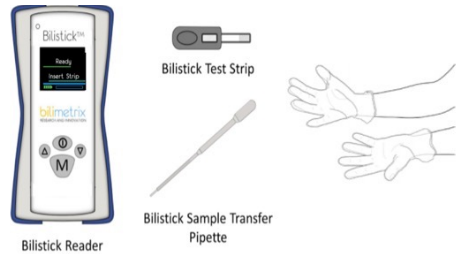

↓

Slow blood flow

or the procedure is interrupted

(baby urinates, baby agitated, ...)

↓

To be within the 2 minutes and 38 seconds time frame after insertion of the strip:

↓

- GENTLY squeeze and release action of the heel to stimulate blood flow

- When the pipette is half full then insert the strip (not earlier)

Picture from the Bilistick user manual and instruction poster

Check the Bilistick strip after the test:

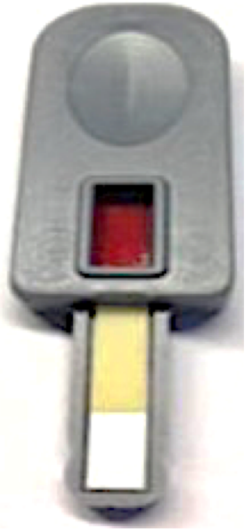

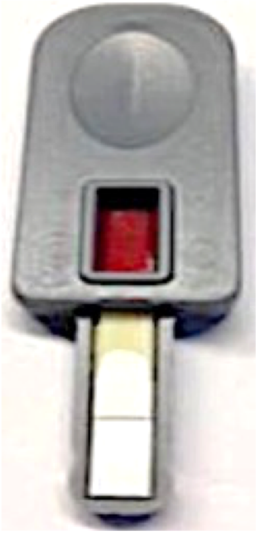

Insufficient saturation of the membrane

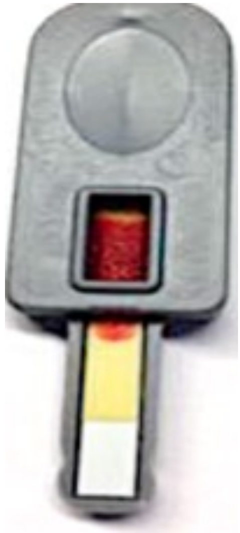

Blood on the membrane

Credit : Laurence Thielemans

Weekly calibration check

When?

- Once a week - Reader will ask for calibration check
- Get the calibration set ready

  (the reader will randomly select one strip from the calibration set)
- Follow the instructions

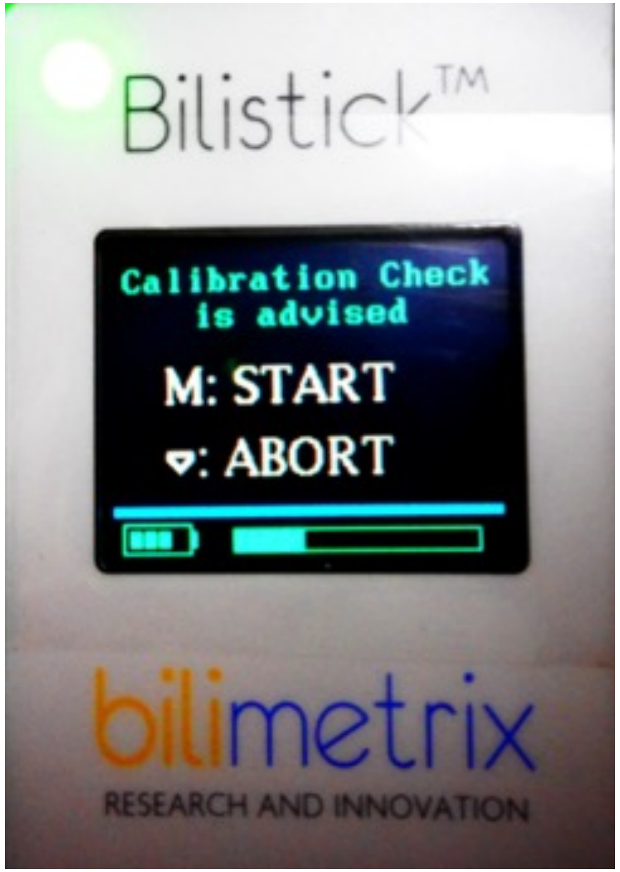

Credit : Laurence Thielemans

Weekly calibration check

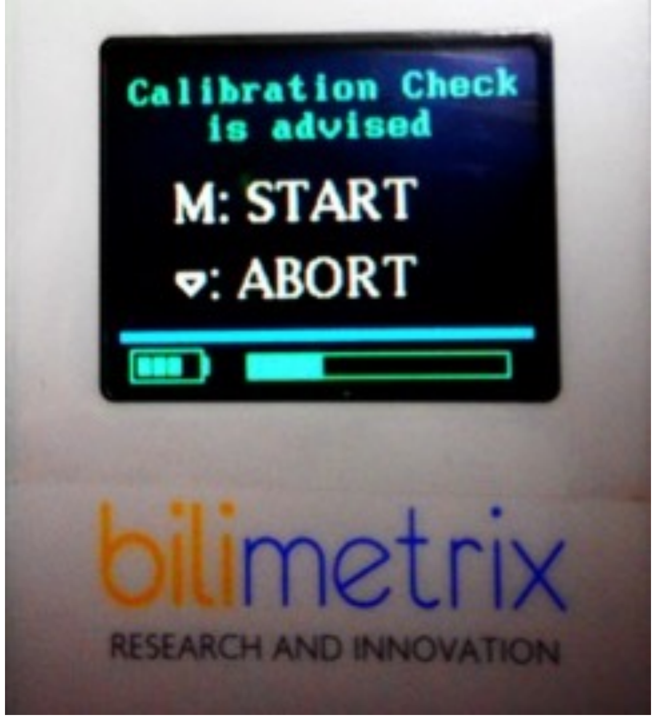

→ Press M →

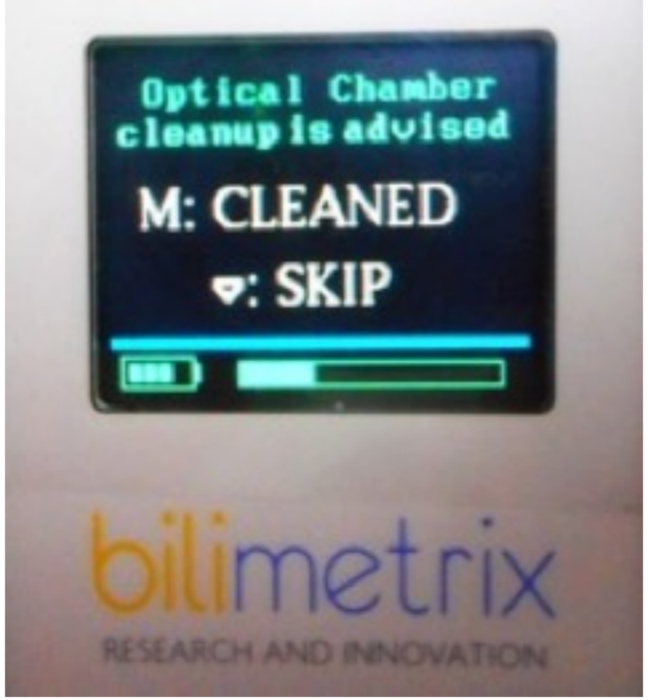

→ Press ▽

Credit Laurence Thielemans

Weekly calibration check

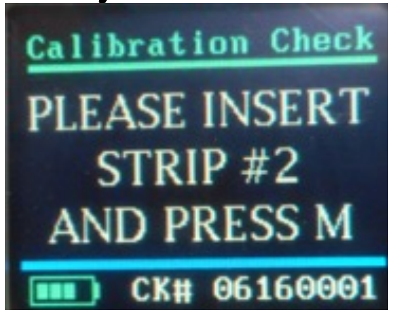

Insert the strip asked by the reader (here nr 2)
Press **M**

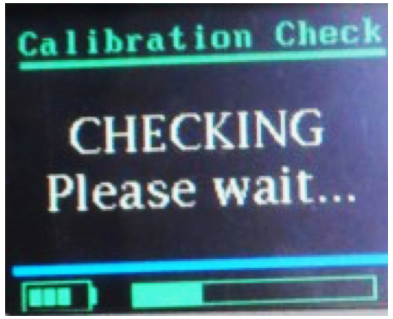

Wait

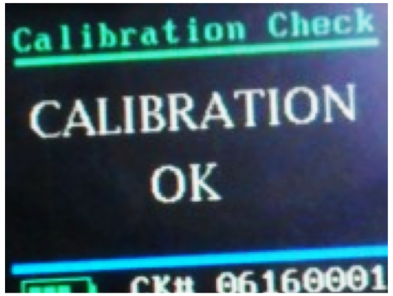

Wait

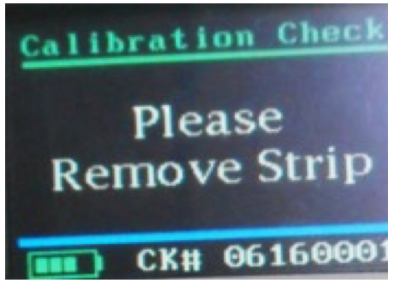

Remove strip

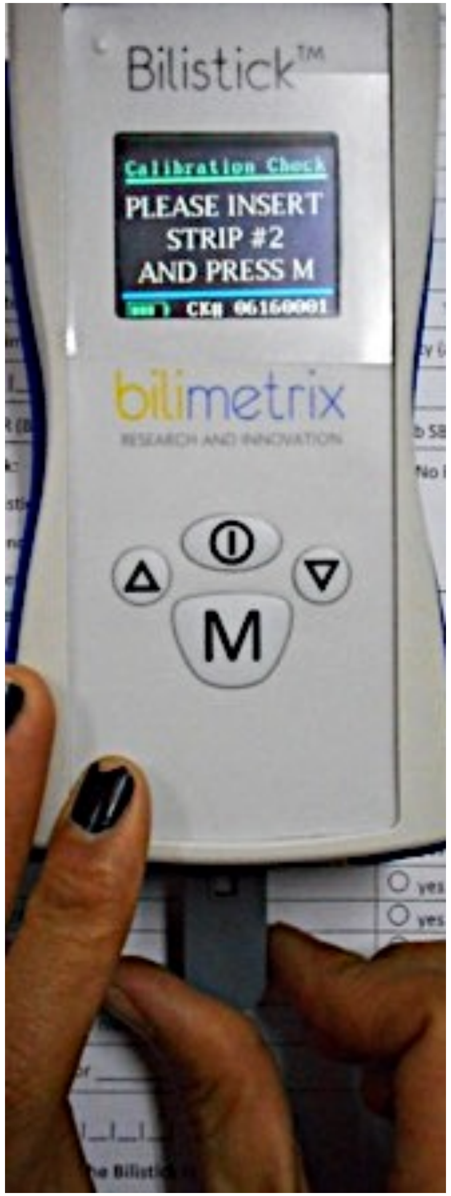

Credit Laurence Thielemans

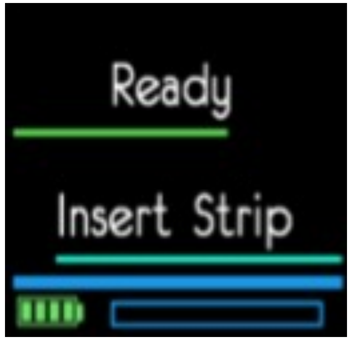

You can do a test or turn off the reader.

Picture from the Bilistick user manual

Weekly calibration check

What if the BS asks for a weekly calibration check but we are ready to perform a test ?

- You can SKIP the calibration and do it later → ABORT (press ▽)
- Do it the next time the BS asks for it

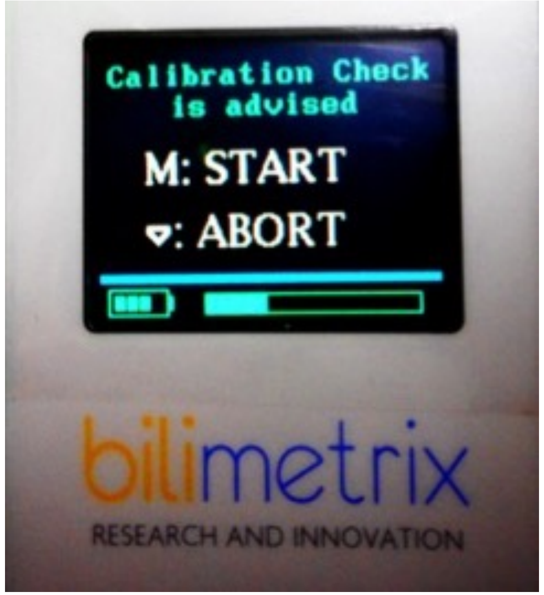

Supplement: Supplementary file 1 [file wellcomeopenres-3-16212-s0008.tgz › 0ecec7f5-0040-412b-8935-61faa07a18dd_New_Supplementary_file_1.pdf]
