# Supplementary figures and images for: Laboratory validation and field usability assessment of a point-of-care test for serum bilirubin levels in neonates in a tropical setting
Source: Wellcome Open Res. 2018 Nov 23;3:110. Originally published 2018 Sep 4. [Version 2] doi: 10.12688/wellcomeopenres.14767.2 (PMC6137410; doi:10.12688/wellcomeopenres.14767.2)

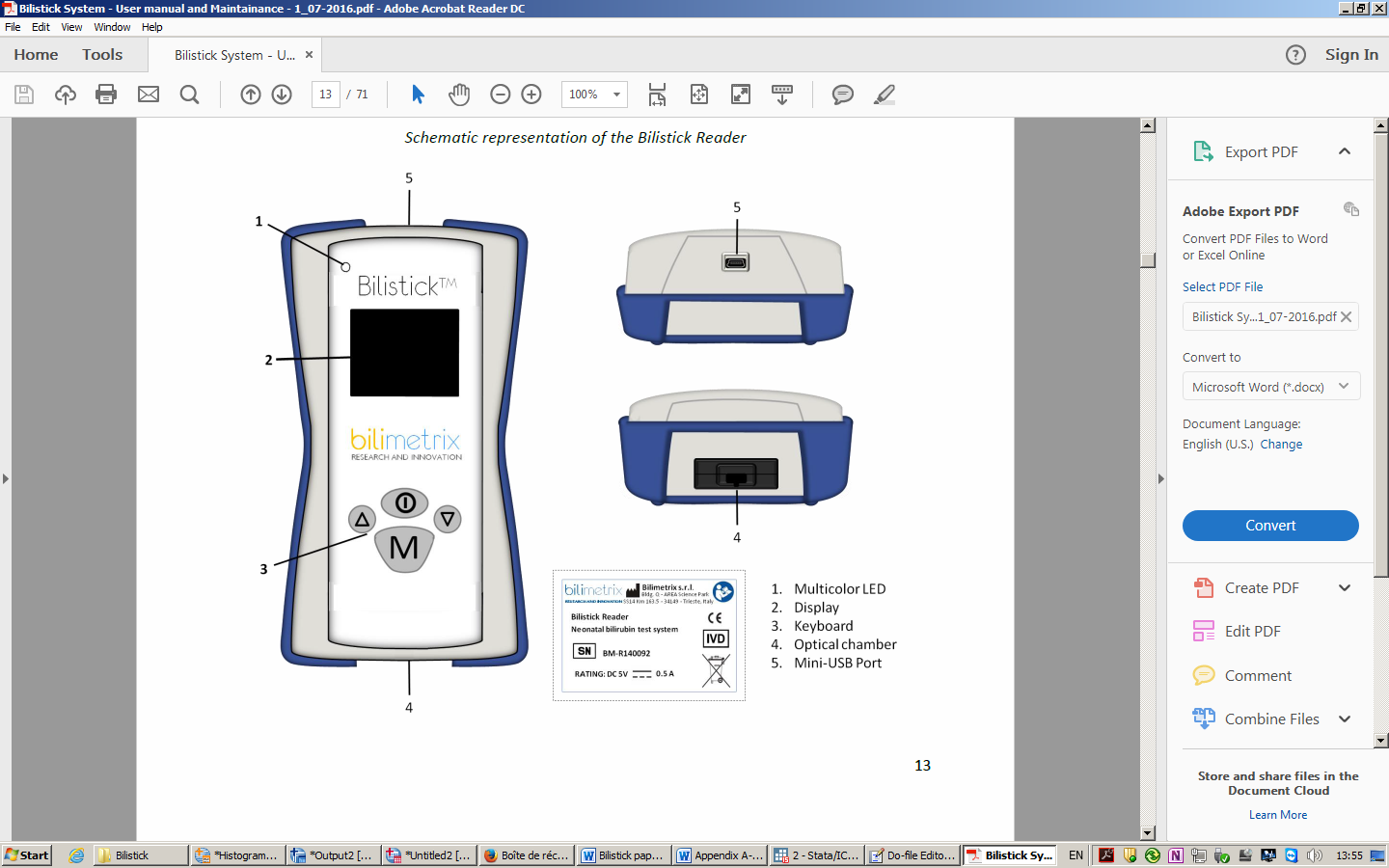

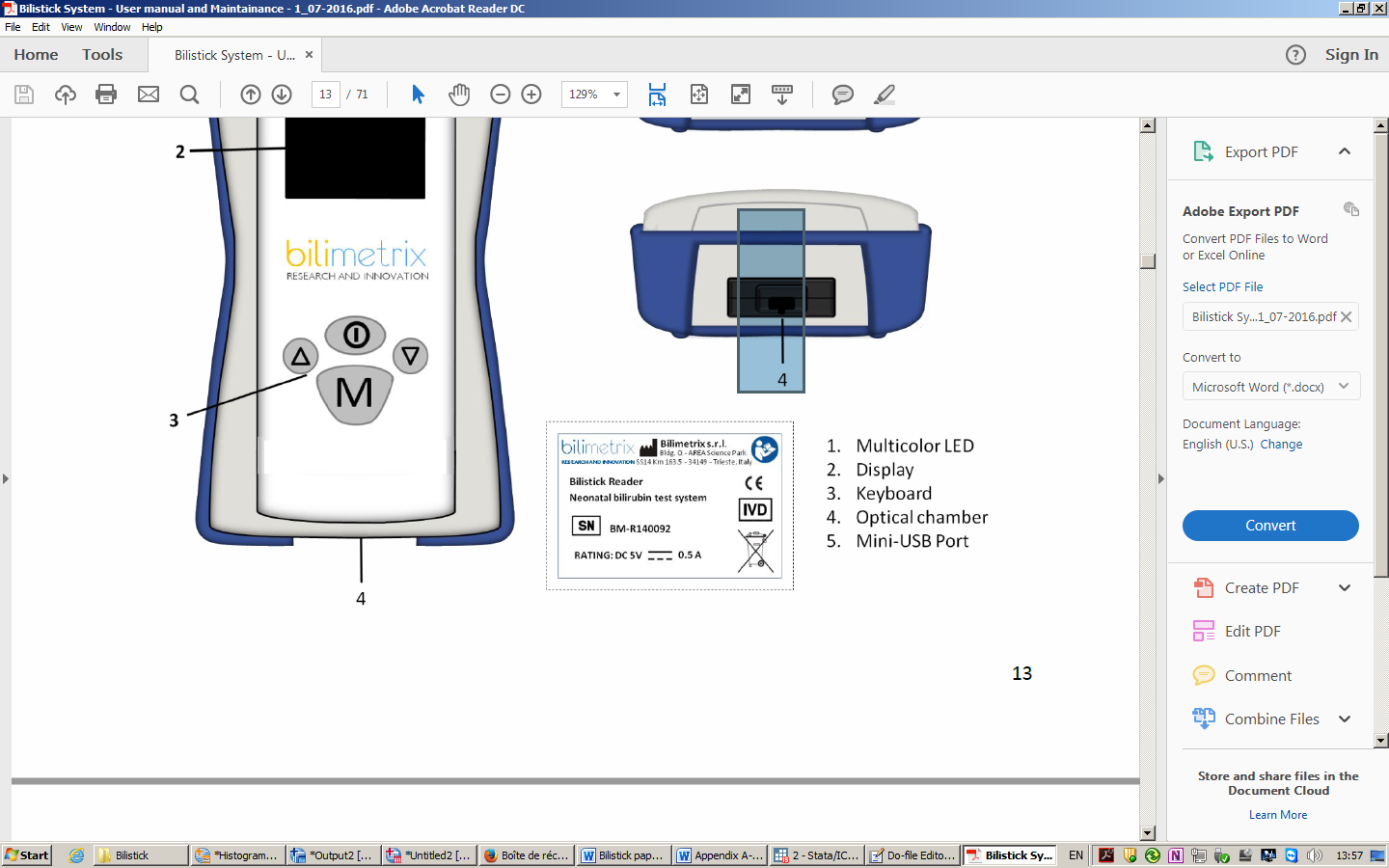

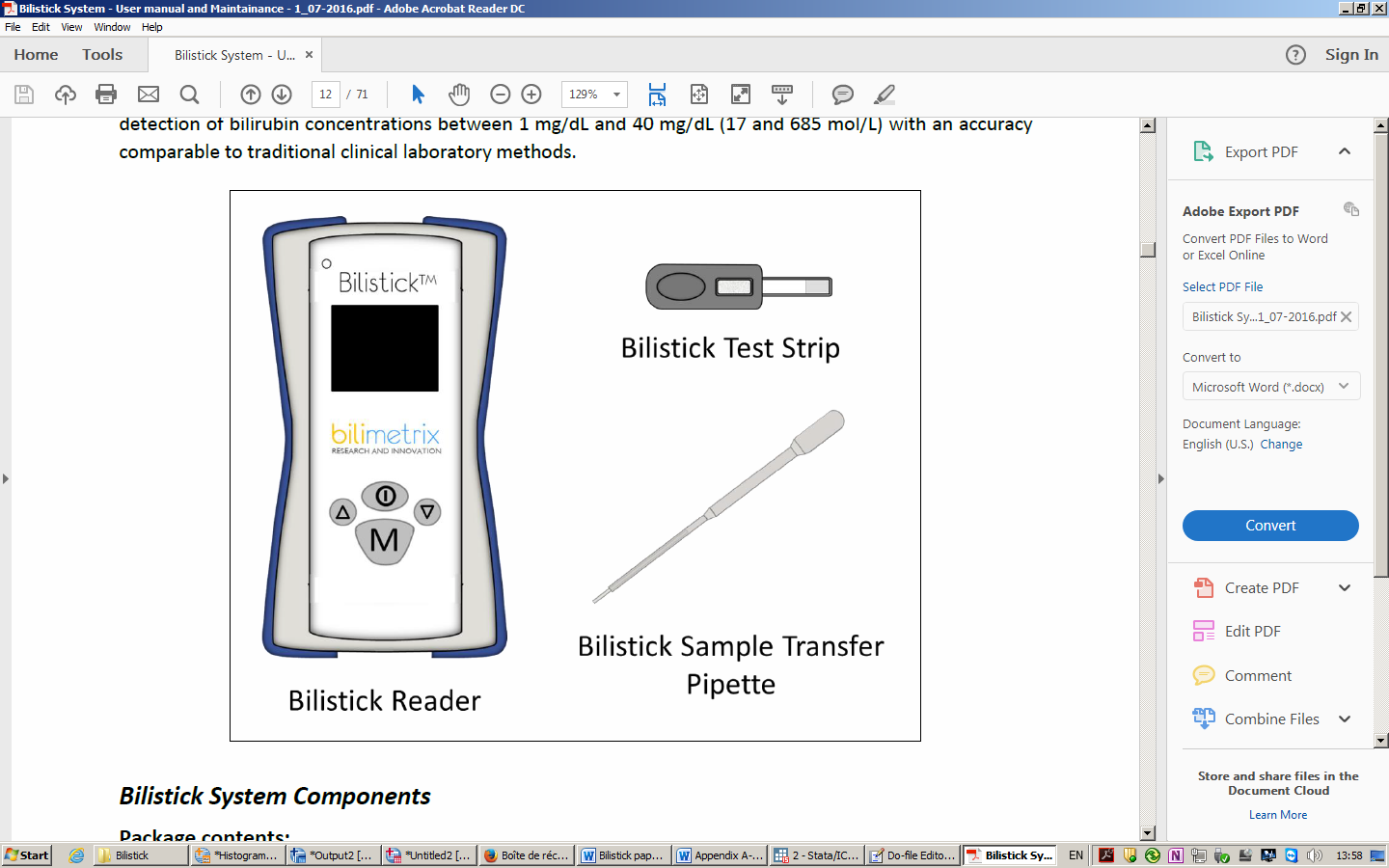

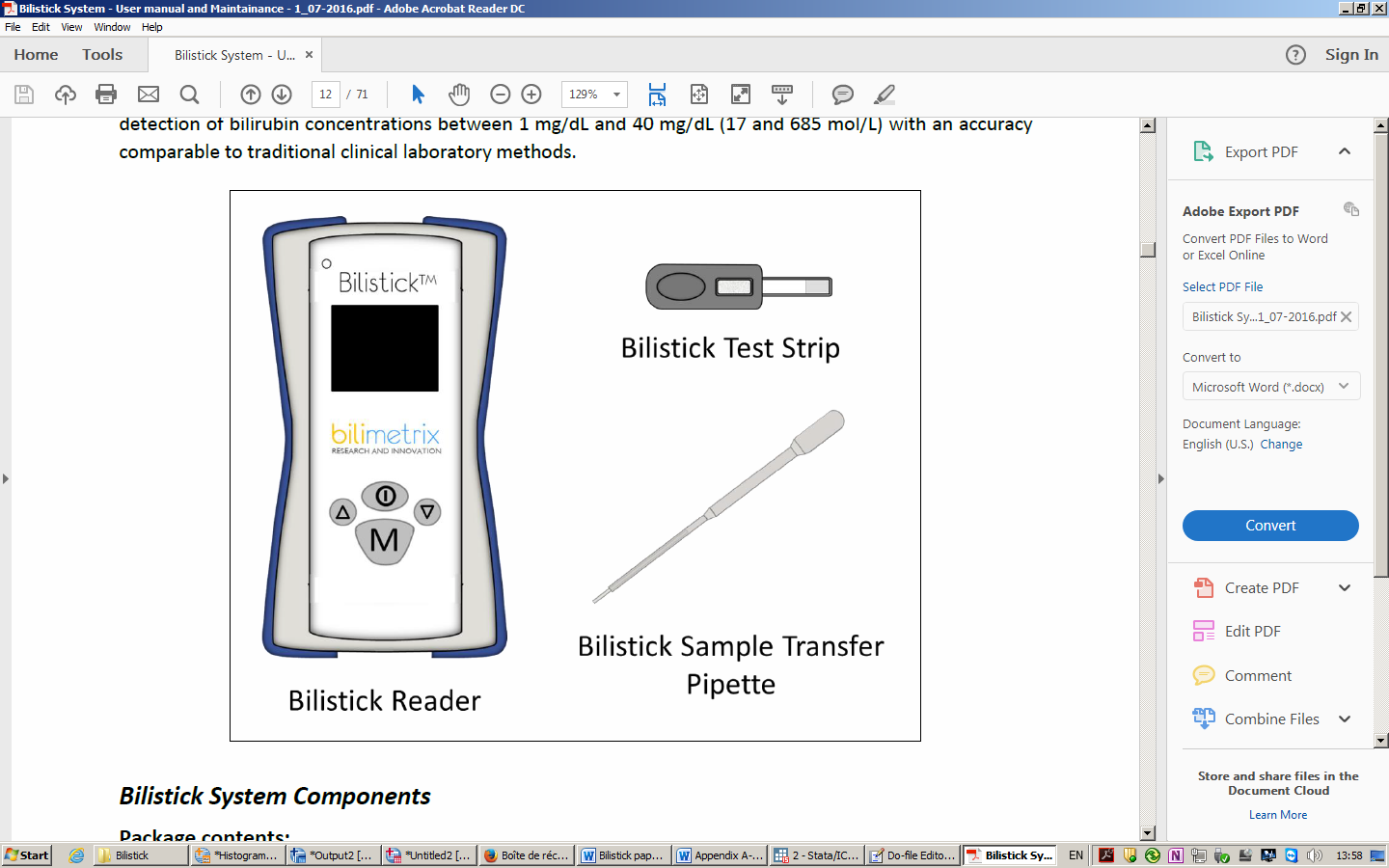

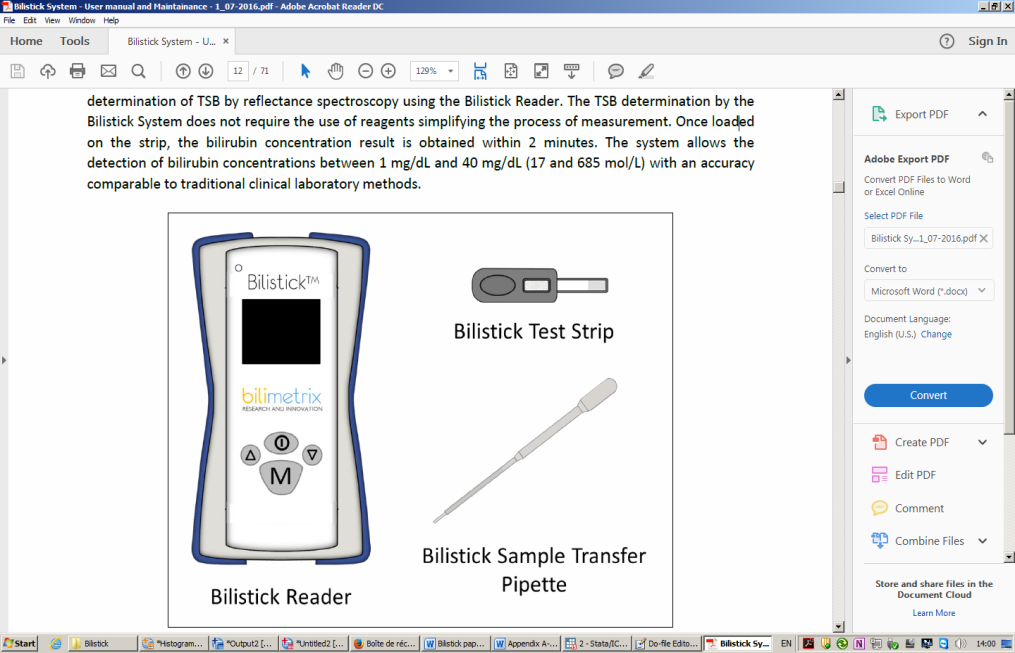


**A1**.


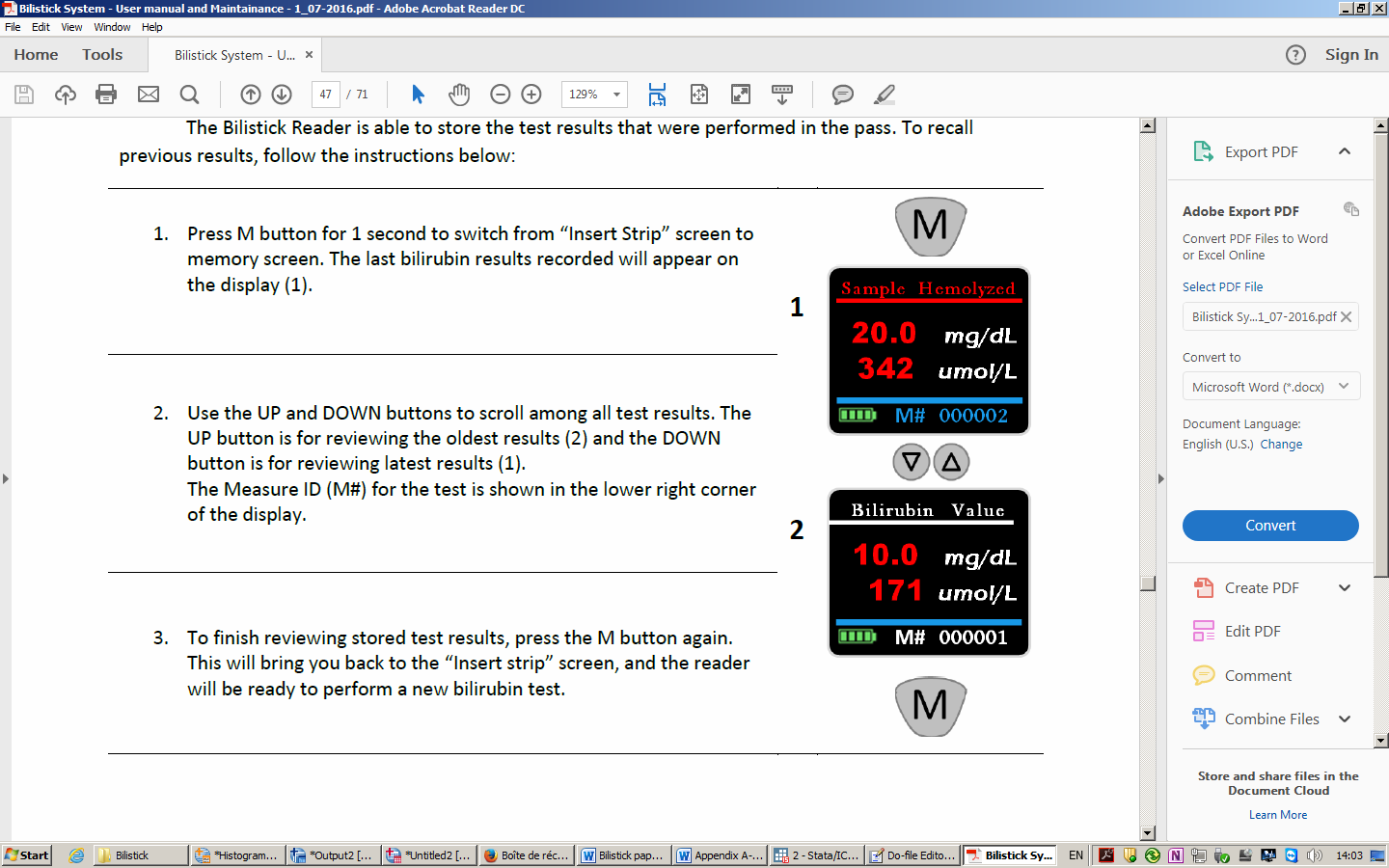
**A2.**

Supplement: Supplementary file 2 [file wellcomeopenres-3-16212-s0000.tgz › 005d155a-e1c5-43cf-bafd-7848c20026da_BS_Supplementary_File_1.docx]
