# Supplementary material for: Laboratory validation and field usability assessment of a point-of-care test for serum bilirubin levels in neonates in a tropical setting
Source: Wellcome Open Res. 2018 Nov 23;3:110. Originally published 2018 Sep 4. [Version 2] doi: 10.12688/wellcomeopenres.14767.2 (PMC6137410; doi:10.12688/wellcomeopenres.14767.2)
Supplement: Supplementary file 3 [file wellcomeopenres-3-16212-s0001.tgz › 5c6f063f-d278-4fd1-b785-c2d35b4fbfb8_BS_Supplementary_File_2.docx]

| **Procedure assessment, n (%)** | **N=173** | **Reader generated error message** |
| --- | --- | --- |
| 1.Touched the strip membrane with the fingers | 0 |  |
| 2.Did not place the reader on a flat surface | 0 |  |
| 3.Inserted the strip into the reader not parallel to the flat surface | 1 (0.6) | 1 |
| 4.Touched or squeeze the bulb while loading the transfer pipette | 0 |  |
| 5.Did not fill the glass portion of the pipette until blood flood stops | 0 |  |
| 6.Squeezed the foot of the baby | 2 (1.2) | 2 |
| 7.Did not squeeze the bulb gently to release the blood | 0 |  |
| 8.Bubbles generated while loading the strip | 9 (5.2) | 7 |
| 9.Touched the membrane with the pipette | 0 |  |
| 10.Did not keep the pressure on the bulb while removing the pipette | 0 |  |
| 11.Did not press the M button | 0 |  |
| 12.Did not remove the strip out of the reader keeping the strip parallel to the table | 0 |  |
