# Supplementary material for: Laboratory validation and field usability assessment of a point-of-care test for serum bilirubin levels in neonates in a tropical setting
Source: Wellcome Open Res. 2018 Nov 23;3:110. Originally published 2018 Sep 4. [Version 2] doi: 10.12688/wellcomeopenres.14767.2 (PMC6137410; doi:10.12688/wellcomeopenres.14767.2)
Supplement: Supplementary file 4 [file wellcomeopenres-3-16212-s0002.tgz › 3c0e0ec1-586a-4a31-969b-8a0f06ebfb9b_supp_file_3.docx]

| Staff ID \|__\|__\|__\| Date: \|__\|__\|__\|__\|__\|__\| |
| --- |


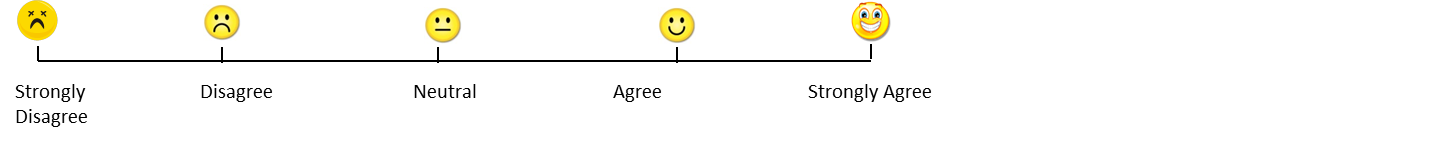


1. **I needed to learn a lot of things before I could get going with the Bilistick system**

Strongly Disagree Disagree Neutral Agree Strongly Agree

1. **The Bilistick system is easy to turn on.**

Strongly Disagree Disagree Neutral Agree Strongly Agree

1. **It is obvious where to place the strip.**

Strongly Disagree Disagree Neutral Agree Strongly Agree

1. **It is easy to place the strip.**

Strongly Disagree Disagree Neutral Agree Strongly Agree

1. **The pipette is easy to use.**

Strongly Disagree Disagree Neutral Agree Strongly Agree

1. **It is obvious** **where to put the blood sample.**

Strongly Disagree Disagree Neutral Agree Strongly Agree

1. **The result is easy to read.**

Strongly Disagree Disagree Neutral Agree Strongly Agree

1. **The Bilistick system is easy to use.**

Strongly Disagree Disagree Neutral Agree Strongly Agree

1. **I need the support of a technical person (lab technician) to be able to use properly the Bilistick system.**

Strongly Disagree Disagree Neutral Agree Strongly Agree

1. **I am able to teach someone else how to use the Bilistick system.**

Strongly Disagree Disagree Neutral Agree Strongly Agree
