# Supplementary material for: Laboratory validation and field usability assessment of a point-of-care test for serum bilirubin levels in neonates in a tropical setting
Source: Wellcome Open Res. 2018 Nov 23;3:110. Originally published 2018 Sep 4. [Version 2] doi: 10.12688/wellcomeopenres.14767.2 (PMC6137410; doi:10.12688/wellcomeopenres.14767.2)
Supplement: Supplementary file 5 [file wellcomeopenres-3-16212-s0003.tgz › d8b89313-ee19-4ba9-9d1b-380192e4cd8e_BS_Supplementary_File_4.docx]

| **Questions** | **Probes** |
| --- | --- |
| What do you think about screening for Jaundice? |  |
| What is the best method to screen for Jaundice? | Kramer zone? Serum bilirubin (SBR)? |
| How do the parents feel about this screening? | Did they have some concerns? What? Why? |
| What is your general opinion about the Bilistick? | What about the test itself? Is it difficult? Time consuming? |
| How did you feel about the training? | Was it good? What can be better next time? Did it prepare you well? |
| If there was no lab support, could you use it for the SBR? | Why? Why not? |
| Do you think this could be used in Myanmar? | By home visitors/at rural health centres/township health centres? Why/why not? |
