# Supplementary material for: Laboratory validation and field usability assessment of a point-of-care test for serum bilirubin levels in neonates in a tropical setting
Source: Wellcome Open Res. 2018 Nov 23;3:110. Originally published 2018 Sep 4. [Version 2] doi: 10.12688/wellcomeopenres.14767.2 (PMC6137410; doi:10.12688/wellcomeopenres.14767.2)
Supplement: Supplementary file 6 [file wellcomeopenres-3-16212-s0005.tgz › 7c19001c-ef2a-4058-963c-c468879f3e3d_BS_Supplementary_File_6.docx]

| **Type errors** | **Description in the user manual** | **Reader#1**  **N=51**  n (%) | **Reader#2**  **N=33**  n (%) | **Visual assessment of the test strip after the test** |
| --- | --- | --- | --- | --- |
| EC:T06 | “*The reading procedure was not completed successfully within the maximum time established by the system, if the error persist control that the haematocrit is within the range accepted by the system (25%-65%)*.” | 33 (64.7) | 31(93.9) | 1 blood clot  11 insufficient test strip saturation  52 normal |
| EC:B03 | “*The system identified an error during the initial phase of the procedure of bilirubin measurement. Probably the M button was not properly pressed*.” | 1* (2.0) | 0 | 1 normal |
| EC:B04 | “*The system identified an error during the bilirubin measurement. A possible cause is that the membrane did not reach the appropriate saturation within the bilirubin test time*.” | 17 (33.3) | 2 (6.1) | 1 blood clot  18 insufficient test strip saturation |

* Error message appears at the time of insertion of the strip. No time to press the M button
