# Supplementary material for: Laboratory validation and field usability assessment of a point-of-care test for serum bilirubin levels in neonates in a tropical setting
Source: Wellcome Open Res. 2018 Nov 23;3:110. Originally published 2018 Sep 4. [Version 2] doi: 10.12688/wellcomeopenres.14767.2 (PMC6137410; doi:10.12688/wellcomeopenres.14767.2)

1.Easy to train

2.Easy to turn on

3.Obvious where to place the strip

4.Easy to place the strip

5.Pipette easy to use

6.Obvious where to place the blood sample

7.Result easy to read

8.Overall easiness of use

9.No need support of a technical person to use the BS

10.Able to teach someone else how to use the BS

0 20 40 60 80 100

(%)


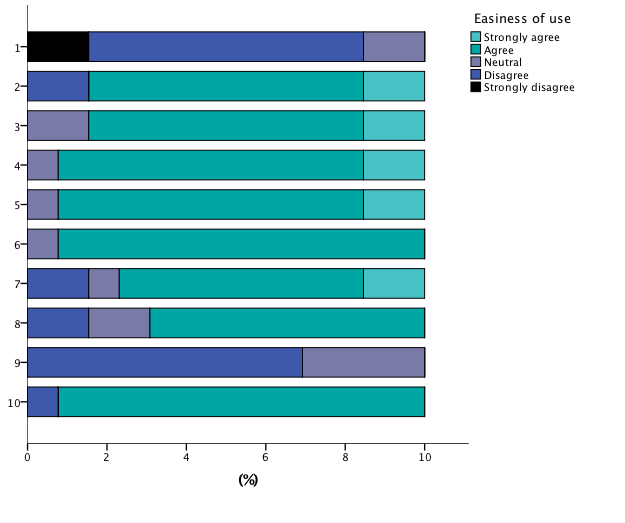

Supplement: Supplementary file 7 [file wellcomeopenres-3-16212-s0006.tgz › f325187c-4816-4aed-91d7-5eb5d7a87d27_Supplementary_File_7.docx]
