# Supplementary material for: Laboratory validation and field usability assessment of a point-of-care test for serum bilirubin levels in neonates in a tropical setting
Source: Wellcome Open Res. 2018 Nov 23;3:110. Originally published 2018 Sep 4. [Version 2] doi: 10.12688/wellcomeopenres.14767.2 (PMC6137410; doi:10.12688/wellcomeopenres.14767.2)
Supplement: Peer review history

# Peer review: Referee response for version 2

**DOI:** 10.21956/wellcomeopenres.16212.r34330

**Published:** 2018-11-26

**Authors:** Bolajoko O Olusanya

**Affiliations:** 1 Centre for Healthy Start Initiative (HSI-Centre), Lagos, Nigeria

The authors have addressed my earlier concerns satisfactorily. This paper is a valuable contribution to the rapidly increasing literature on Bilistick and other POC devices for bilirubin determination.

I have read this submission. I believe that I have an appropriate level of expertise to confirm that it is of an acceptable scientific standard.

# Peer review: Referee response for version 1

**DOI:** 10.21956/wellcomeopenres.16093.r33822

**Published:** 2018-09-21

**Authors:** Bolajoko O Olusanya

**Affiliations:** 1 Centre for Healthy Start Initiative (HSI-Centre), Lagos, Nigeria

**General**

 The authors of this paper rightly recognised the need for reliable, cost-effective and easy to use point of care (POC) bilirubin measuring devices in developing countries. They set out to determine the reliability and usability of a newly developed POC device, Bilistick System (BS) at a field clinic along the Thailand-Myanmar border region using a mixed-methods approach from January to December 2017. The main findings were that the BS did not produce usable results in almost half of the tests but was found to be easy-to-use by the midwives. The authors thus concluded that BS was easy-to-use but not reliable for measuring serum bilirubin in a low-resource setting.

**Specific issues to address**

 The introduction to the study and the references cited throughout the paper clearly reflect the authors’ lack of familiarity with the relevant studies from low and middle-income countries (LMICs). For example, the authors only recognised that TCB underestimates serum bilirubin whereas there is preponderance of evidence that TCB more commonly overestimates serum bilirubin in special populations in LMICs. Studies that seek to address needs in LMICs must acknowledge and build on the body of evidence from the region to be considered as a credible and valuable addition to knowledge.

 Generally, the methodology is satisfactory. However, the authors need to clarify if the study participants were all in-born as the article seems to suggest. How were the out-born neonates managed? How was gestational age determined in these babies? How much did it cost to test a neonate using BS?

 The conclusions are valid. BS in its current form does not appear to provide any benefit over the non-invasive TCB devices as its reliability diminishes with increasing haematocrit level above 55%. Perhaps, more crucially, the study has highlighted a major drawback and context for evaluating a recent multi-centre study ^1^ that seems to have exaggerated the utility of BS in developing countries. Whereas Greco et al reported technical problems in only 6.8% of the tests, the current study showed a significantly higher percentage (48.6%) of error messages. Evidently, improvements in the BS are warranted.

 The study reported that the 82 paired results from BS and bilirubinometer were analysed. The authors however, reported that a limitation of the study was that BS was not compared to the gold standard. The authors need to clarify which gold standard is required other than serum bilirubin.

 Abbreviations used should be summarised at the end of the manuscript.

I have read this submission. I believe that I have an appropriate level of expertise to confirm that it is of an acceptable scientific standard, however I have significant reservations, as outlined above.

## References

[1] GrecoCIskanderIEl HouchiSRohsiswatmoRRundjanLOgalaWOfakunrinAMocciaLHoiNBedogniGTiribelliCCoda ZabettaC: Diagnostic Performance Analysis of the Point-of-Care Bilistick System in Identifying Severe Neonatal Hyperbilirubinemia by a Multi-Country Approach. *EClinicalMedicine*.2018;1: 10.1016/j.eclinm.2018.06.003 14-20 10.1016/j.eclinm.2018.06.003 PMC653756331193593

# Response

**Published:** 2018-11-07

**Authors:** Laurence Thielemans

**Affiliations:** 1Shoklo Malaria Research Unit, Mahidol-Oxford Tropical Medicine Research Unit, Faculty of Tropical Medicine, Mahidol University, Mae Sot, Thailand

Please find below our responses to the comments of the reviewer.

1. The introduction has been modified as suggested, including additional references to TCB performances in LMICs.

2.1. Being born outside SMRU clinics was not an exclusion criterion. Mothers who delivered outside (i.e. at home) were advised to visit SMRU clinics as soon as possible after the delivery and all neonates were managed in the same way at the clinics.

2.2. Gestational age was routinely estimated either by ultrasound at the first antenatal consultation (ANC) or by Dubowitz gestational assessment at birth. The information was added in the methods and results.

2.3 We did not perform a formal cost analysis for testing BS in our setting. For the study, the Bilistick system was lent by Bilimetrix together with 200 strips and pipettes.Information was added in the methods.

3. For assessment of serum bilirubin, the laboratory gold standard is analysis by high performance liquid chromatography (HPLC, Kazmierczak et al., 2002) but spectrophotometric assays are more commonly used and considered reliable as reference tests. For this study the serum bilirubin was measured with the dual-wavelength BR-501 bilirubinometer (Apel Co., Ldt, Japan). We have clarified this point in the discussion. 

4. A summary of the used abbreviations has been added at the end of the manuscript as suggested

# Peer review: Referee response for version 1

**DOI:** 10.21956/wellcomeopenres.16093.r33830

**Published:** 2018-09-12

**Authors:** Robert D Christensen

**Affiliations:** 1 Division of Neonatology, University of Utah, Salt Lake City, UT, USA

Thielemans *et al.* report a prospective investigation of the feasibility and diagnostic accuracy of a potential method for quantifying serum bilirubin in a tropical, low-resource, setting. The device is called the Bilistick System, and involves a small quantity of blood, typically drawn by heel-stick, and run as in a battery-operated, point of care system. These features would render this method far more practical and economical than standard laboratory-based serum bilirubin testing.

 Severe hyperbilirubinemia that goes undetected and untreated can lead to acute bilirubin encephalopathy, when bilirubin crosses from blood to brain and damages, sometimes permanently, the central nervous system. The consequent death or life-long handicapping conditions, often consisting of deafness and athetoid cerebral palsy, place a large burden on families and societies. This tragic condition is one of the most heart-wrenching medical safety concerns in global pediatrics, because the damage is, in theory, completely preventable with early recognition and treatment of neonatal hyperbilirubinemia.

 The device did not test sufficiently well as to recommend its use in present configuration. However, the study appears to have been well conceived and executed and may give others the focus needed to improve the performance of this system, particularly in conditions with hematocrits above 55% and humidity condition above about 75%.

I have read this submission. I believe that I have an appropriate level of expertise to confirm that it is of an acceptable scientific standard.

# Peer review: Referee response for version 1

**DOI:** 10.21956/wellcomeopenres.16093.r33824

**Published:** 2018-09-11

**Authors:** Rebecca R. Richards-Kortum, Meaghan Bond

**Affiliations:** 1 Department of Bioengineering, Rice University, Houston, TX, USA; 2 Rice University, Houston, TX, USA

This interesting paper describes a well-designed study to evaluate the accuracy and usability of a new point-of-care test to measure total serum bilirubin in low-resource settings.The results indicate an unacceptably high number of errors, despite observation of target users that indicated the test was being performed properly. The results will be of interest to researchers in the global health and the diagnostics communities. In particular, the paper highlights previously unreported challenges associated with achieving accurate results in high humidity and for neonates with high hematocrit levels.

 The authors are encouraged to consider a number of minor questions and comments that would be of interest or helpful to readers: 

1. Please include the author-developed training materials as Supplemental Information. What are the primary differences between the manufacturer and the author-developed training materials? Could this have affected results?
2. The authors state that “Since the BS required a haematocrit (HCT) ≤65% to provide a reading, neonates with a prior HCT >65% or no prior HCT measured were not eligible.”
  1. How many babies does this exclude?
  2. Why were babies with no prior HCT measured not eligible, since HCT was measured for the 50 uL gold standard blood sample?
3. It would be helpful to clarify the calibration process for the device. The methods state that the calibration was “checked weekly,” but also that the user must insert a strip a maximum of 2 minutes before each sample for calibration.
4. To facilitate comparison of data from the two readers, it would be more helpful to put data from both readers on same graph with different symbols used for each.
5. It would be helpful to clarify earlier in paper that clotting due to delays occurs in the non-heparinized blood collection pipette, not on the BS strip.
6. The data in supplemental 5 (details about malfunctions and the manufacturer’s attempts to fix them) seem important for real-world use and for understanding the high percentage of error messages. Could the authors move this paragraph to the main body of the paper.
7. The observations of how the test was used in practice are interesting. Could the authors briefly describe how the 2-minute time limit between strip calibration and sample measurement monitored. Is this actively measured by the BiliStick device? If not, how can the user tell that 2 minutes has passed and the sample is no longer accurate?
8. The data in Figure 2 show differences in the performance of the two readers; based on data in Tables 3 and 4, these differences are attributed to differences in the humidity at the time of use and the fraction of samples with high HCT measured using reader 2. Are there sufficient data to allow the authors to compare results for Readers 1 and 2 under the ideal conditions of humidity <75% and HCT between 30-45% and assess whether there are any remaining reader-to-reader variations?

We have read this submission. We believe that we have an appropriate level of expertise to confirm that it is of an acceptable scientific standard.

# Response

**Published:** 2018-11-07

**Authors:** Laurence Thielemans

**Affiliations:** 1Shoklo Malaria Research Unit, Mahidol-Oxford Tropical Medicine Research Unit, Faculty of Tropical Medicine, Mahidol University, Mae Sot, Thailand

We appreciate the comments of the reviewer Rebecca R. Richards-Kortum. Please find below our Point-by-point answers.

 1.The training material has been added as a supplementary file as suggested. The manufacturer provided a user manual, a user instruction poster and a video in English. However they were too difficult (and the video also too rapid) to understand for non-native English speaking staff and the instruction poster and user manual contained an error (acknowledged by the manufacturer). A simplified power point presentation was used instead, explaining the procedure step-by-step, highlighting the importance of timing at each step (turning the device on in time, waiting for the reader to display an instruction, inserting the strip in time etc.). Additional topics were addressed such as how to load the pipette correctly, how to deal with time constraints or a slow blood flow, and how to check the BS strip after the test. The required weekly calibration procedure was also explained. The video was replaced by a step-by-step demonstration on-site.

 As we did not perform a training using the original material it is not possible to quantify or assess the impact but it is unlikely that adapting the training material affected the results negatively; indeed simplified explanations and live demonstration are usually expected to improve user understanding of procedures. The content of the training was the same as the instruction manual and poster, only its form was adapted. In essence, this was a process of tailoring instructions for appropriate BS use to the local context.  

 2.1. We did not record the number of non-eligible neonates in this study. We can estimate that around 20-25% of children seen within 24h of life might have been excluded in this setting, based on results from a recently completed analysis birth cohort (ref 29 in manuscript doi:  10.1186/s12887-017-0798-8), where capillary Hct was measured routinely between 12-30h of life; the proportion of neonates with capillary Hct >65% was 17.4% (193/1111) and with capillary Hct ≥70% 8.4% (93/1111) (unpublished data). We have added a sentence in the discussion in line with this (see paragraph 4, last sentence).  

 2.2. The Hct reading from the standard blood sample was not readily available (due to a 30 minute delay) and we did not want to heel prick the neonates twice. This, combined with a high proportion of neonates with Hct >65% and a small number of available strips (only 200 for a sample size of 180), led to the decision to maximize the number of valid readings and thus exclude babies without prior known Hct values.

 3. We have now clarified the complete calibration process of the BS device in the methods.

 4. Figure 2 was modified as recommended.

 5. We have now clarified this in the methods.

 6. The paragraph has been added to the main manuscript (in the  result: technical support) as suggested.

 7. The 2-minute time limit was calculated by the Bilistick device itself and a warning message was displayed once the time limit was reached so that a new strip had to be inserted. We clarified the process in the methods.

 8. The analysable data performed under the ideal conditions of humidity <75% and HCT 30-55% are limited to 30 and 15 tests with Reader #1 and Reader #2, respectively. Pearson correlation coefficient was similar in both groups (r=0.97 (95% CI: 0.93–0.98) and r=0.98 (95% CI: 0.94–0.99)). The information has been added in the results.
